# An automated approach for the identification of horizontal gene transfers from complete genomes reveals the rhizome of Rickettsiales

**DOI:** 10.1186/1471-2148-12-243

**Published:** 2012-12-12

**Authors:** Phuong Thi Le, Hemalatha Golaconda Ramulu, Laurent Guijarro, Julien Paganini, Philippe Gouret, Olivier Chabrol, Dider Raoult, Pierre Pontarotti

**Affiliations:** 1 Evolutionary biology and modeling, LATP UMR-CNRS 7353, Aix-Marseille University, Marseille, 13331, France; 2 Unit for Research on Emergent and Tropical Infectious Diseases, URMITE UMR CNRS 7278, IRD 198, Inserm 1095, Aix-Marseille University, Marseille, 13005, France

**Keywords:** Horizontal gene transfer, *Rickettsiales*, *Candidatus* Pelagibacter ubique, Sympatry

## Abstract

**Background:**

Horizontal gene transfer (HGT) is considered to be a major force driving the evolutionary history of prokaryotes. HGT is widespread in prokaryotes, contributing to the genomic repertoire of prokaryotic organisms, and is particularly apparent in *Rickettsiales* genomes. Gene gains from both distantly and closely related organisms play crucial roles in the evolution of bacterial genomes. In this work, we focus on genes transferred from distantly related species into *Rickettsiales* species.

**Results:**

We developed an automated approach for the detection of HGT from other organisms (excluding alphaproteobacteria) into *Rickettsiales* genomes. Our systematic approach consisted of several specialized features including the application of a parsimony method for inferring phyletic patterns followed by blast filter, automated phylogenetic reconstruction and the application of patterns for HGT detection. We identified 42 instances of HGT in 31 complete *Rickettsiales* genomes, of which 38 were previously unidentified instances of HGT from *Anaplasma*, *Wolbachia*, *Candidatus* Pelagibacter ubique and *Rickettsia* genomes. Additionally, putative cases with no phylogenetic support were assigned gene ontology terms. Overall, these transfers could be characterized as “rhizome-like”.

**Conclusions:**

Our analysis provides a comprehensive, systematic approach for the automated detection of HGTs from several complete proteome sequences that can be applied to detect instances of HGT within other genomes of interest.

## Background

Horizontal gene transfer (HGT) has played a significant role in bacterial evolution [1]. HGT is widespread in prokaryotes [2] and is considered to be a driving force in the innovation and evolution of bacterial genomes [3,4]. HGTs have been shown to contribute to the genomic repertoires of a number of prokaryotes, including *Rickettsiales* species.

The order *Rickettsiales* has been classified into two families, *Rickettsiaceae* and *Anaplasmataceae*. Members of the *Rickettsiales* order are primarily intracellular bacteria and can encompass both parasitic genera, such as *Rickettsia* and *Orientia*, or symbiotic genera, such as *Wolbachia*. Over the course of their evolution, the intracellular lifestyle of these members has led to the irreversible loss of genes [5,6]. *Orientia* species cause scrub typhus in human and are classified in a separate clade from *Rickettsiae* species [7-9]. *Rickettsiae* species are associated with a diverse host range that include vertebrates, arthropods, annelids, amoebae and plants and are known as arthropod-vector pathogens of vertebrate hosts [10,11]. The family *Anaplasmataceae* encompasses the three genera *Anaplasma*, *Ehrlichia* and *Wolbachia*[12]. *A. phagocytophilum* causes human granulocytic ehrlichiosis [13], *E. chaffensis* causes human monocytic ehrlichiosis [14,15] and *Neorickettsia sennetsu* causes sennetsu ehrlichiosis, an infectious mononucleosis-like disease [16]. The *Wolbachia* species live as symbionts in arthropods and annelids. The genomes of these species demonstrate both genome reduction and gene integration events between symbionts and host nuclear genomes [17]. *Candidatus* Pelagibacter ubique is a marine, free-living bacterium with a small, AT-rich genome that belongs to the SAR11 clade [18]. *Candidatus* Pelagibacter ubique was included under the sister group of the *Rickettsiales* clade in 2007 [19], but the placement of this free-living bacterium in an obligate intracellular phylum remains a subject of debate [20-24].

HGTs have occurred at high frequencies within alphaproteobacteria species [25] and within the *Rickettsia*[26]; therefore, we limited our analysis to the study of genes transferred from distantly related species into *Rickettsiales* species genomes. Previously, we have reported the existence of novel HGTs from a number of other genomes into *Rickettsiales* species [27-29]. In the current study, we have detected these new instances of transfer by developing an automated approach for the detection of HGT from complete proteomes.

Currently, a number of methods exist for detecting HGTs [30-40]. The two main approaches depend on the analysis of sequence composition and the use of phylogenetic methods. The first examines atypical GC content, codon usage bias, oligonucleotide frequencies, and genomic signatures [30-34]. The second enables the detection of inconsistencies in gene and genome evolutionary history either (i) by reconstructing the gene tree and comparing it with the reference species tree, i.e., via the test of topologies, bipartition, quartet bipartitions [35-37]; or (ii) by examining gene history, i.e., phyletic profiles [38,39]. We herein describe a new strategy for systematically searching for instances of HGT that depends on the use of stringent filters. Using this method, a cluster of orthologous protein sequences were first retrieved and examinated for sequence similarity with the organisms of interest. This was followed by the application of phyletic pattern detection for the identification of gain events. The results were then analyzed by Blast searches to retrieve sequences that were homologous to *Rickettsiales* genomes from other organisms, excluding alphaproteobacteria. These results were then examinated by automated phylogenetic analysis, followed by the detection of instances of HGT. Using this method, we were also able to verify the HGT events reported by previous studies. Here, we provide a detailed description of the transferred proteins, including their predicted function and ecological niches, as well as an analysis of the function of putative transfers with no phylogenetic support.

## Results

### Clustered Ortholog Groups (COGs)

We analyzed 38,895 proteins from 31 complete proteomes using OrthoMCL, of which 1,594 (4%) proteins were found to be less than 50 amino acids in length and therefore discarded. From the remaining 37,301 (96%) proteins, 4,240 (11%) belonged to non-orthologous groups that did not form clusters, and 33,061 (85%) were clustered into 3,537 orthologous groups. We found that 267 (7.6%) such Clustered Ortholog Groups (COGs) were conserved between *Caulobacter cresentus* CB15 and *Rickettsiales* species that were among the 31 species included in our study (Additional file 1, Table S3; Additional file 2, Figure S10). An additional 277 COGs were conserved among *Rickettsia*, *Orientia*, *Wolbachia*, *Anaplasma*, *Ehrlichia*, *Neorickettsia*, and *Candidatus* Pelagibacter ubique genome (Additional file 1, Table S4; Additional file 2, Figure S10), and 328 COGs were conserved among *Rickettsia*, *Orientia*, *Wolbachia*, *Anaplasma*, *Ehrlichia*, *Neorickettsia* genomes (Additional file 1, Table S5). We found 549 conserved COGs among *Wolbachia*, *Anaplasma*, *Ehrlichia*, and *Neorickettsia* genomes, and 497 conserved COGs were found only in *Rickettsia* and *Orientia* genomes. We also carried out functional analyses for these orthologous groups using BlastP against the COG database [41,42] with an E-value cutoff of less than 10-10 and annotated 1,709 (48.5%) orthologous groups. The results of these analyses are provided in Additional file 1, Table S6 and Additional file 2, Figure S10.

### Detection of gain and loss events from 3,537 COGs

**Figure 1:**
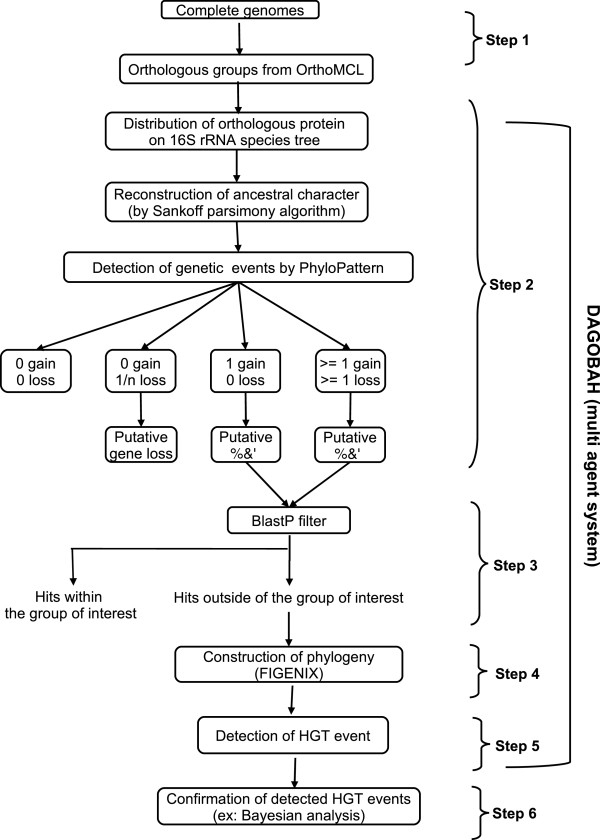
**Flowchart representing the systematic pipeline for the automated detection of horizontal gene transfer in complete genomes.** Step 1. Clustering of orthologous groups by OrthoMCL. Step 2. Detection of the presence or absence of genetic events on the species tree and the reconstruction of the phyletic pattern of gene gain and gene loss using PhyloPattern. Step 3. BlastP filter to retrieve the homologous sequences. Step 4. Construction of phylogenetic trees using FIGENIX. Step 5. Detection of HGT events from HGT agent using a transfer filter. Step 6. Validation of HGT cases using Bayesian method. Steps 1 through 5 are included in the pipeline.

To characterize gains and losses, the presence and absence of genes from the species trees was mapped onto orthologous groups, and phyletic patterns of gene gain and gene loss events were reconstructed. The results thus obtained from pattern detection were logically classified into four types in the following manner: 0 gain, 0 loss; 0 gain, 1 loss; 1 gain, 0 loss; and at least 1 gain 1 loss (Figure 1). Accordingly, the results from each orthologous group that fell under each type were analyzed for gain and loss events. In this way, we obtained 267 COGs with 0 gain and 0 loss events, 752 COGs with 0 gain and 1 loss, and 2,518 COGs with at least 1 gain and 1 loss event (Additional file 2, Figure S5). As mentioned previously, we evaluated only instances of gain events due to HGT, which formed the main objective of our study.

### Analysis of HGT cases from only gain event

The 2,518 COGs with at least 1 gain event were automatically identified using Blast filter, phylogenetic analysis, and HGT detection and were validated by a human expert using a Bayesian approach as described in the methods. We found that 838 (33.3%) proteins had hits outside of alphaproteobacterial species, while the remaining 1,680 (66.7%) proteins had hits within alphaproteobacterial species (Additional file 2, Figure S6a). The 838 proteins were subjected to automated phylogenetic reconstruction and HGT detection. Phylogenetic trees were successfully reconstructed for 56.9% of the cases, out of which 4.3% (36 cases) were associated with strong bootstrap support for HGT, while the remaining 52.2% were weakly supported by bootstrap values. The remaining 43.5% of cases failed to produce phylogenetic trees based on the application of stringenet FIGENIX parameters, which are listed as follows: gap sizes that exceeded the threshold by more than 30%; the presence of a greater number of repeats; an insufficient number of Blast hits that did not allow for phylogenetic reconstruction; and detection by puzzle filter of quickly evolving sites resulting from the duplication of paralogous groups that hindered phylogenetic reconstruction. The corresponding genes may be considered to be putative cases of HGT but with weak or no phylogenetic support. We will further describe the distribution of these genes based on Gene Ontology (GO) database [43] in order to classify their putative functions.

Finally, we found 4.3% of proteins (36 cases) with phylogenetic support for HGT with at least 1 gain event, of which 12 cases were detected from uncultured bacteria to *Candidatus* Pelagibacter ubique (data not shown) and were therefore excluded from further analysis. The remaining 24 cases were found to be good candidates for HGT from different organisms with well-supported bootstrap values. Of these 24 cases, two have been reported as HGTs in previous studies, gi:42520378 was reported as to be an instance of HGT from a *Wolbachia* strain to *Aedes aegypti* by Klasson et al., 2009 [44] and gi:190570783 was reported to be an instance of HGT from Wolbachia phage WO into *Wolbachia* species by Wu et al., 2004 [45] (Additional file 1, Table S8). The remaining 22 instances of HGTs were present in *Anaplasma*, *Wolbachia* and *Rickettsia* species genomes. These proteins were acquired from different origins, including bacterial species from *Bacteroidetes*, *Firmicutes*, *Chlamydia*, *Spirochaetales*, *Nitrospirales*, and *Proteobacteria* clades (Beta, Gamma and Delta). The functions of these transferred proteins are diverse, including ATPases, aldolases, transporter activities, cystathionine beta-lyases, sugar phosphate permeases, growth inhibitors and antitoxin activities, while the others were found to be hypothetical with no known function.

To determine the possible origin of these hypothetical proteins, we analyzed them using the ACLAME database. We report here only the best match, which was for gi:238651161 with plasmid:126745 from *Rickettsia felis* URRWXCal2, with an E-value of 3.5e-27 and 69.5% identity. The transfer of this gene (gi:238651161) was from *Actinobacillus minor* NB305 to *R. felis* and *R. peacockii* str. Rustic (Additional file 1, Table S8).

### HGT cases from non-orthologous groups

We applied a similar procedure to the 4,240 non-orthologous proteins to detect transfer events. Of these, 1,795 proteins shared with *Caulobacter cresentus* CB15 and were therefore discarded from further analysis. The remaining 2,445 proteins were analyzed by BlastP against the NR database with a cutoff of E-value of 10-10. Of these, 680 (27.8%) proteins had hits outside of the alphaproteobacteria species, and 1,765 (72.2%) proteins had hits within alphaproteobacterial species (Additional file 2, Figure S6b). The 680 proteins were further analyzed for HGT in a similar manner to that discussed above. Phylogenetic trees were reconstructed for 61.6% of the cases, of which 53% were weakly supported and the remaining 8.6% (59 cases) were strongly supported by bootstrap values. The remaining 38.4% of cases did not result in phylogeny due to the reasons previously discussed. Thus, we identified 59 instances of HGT from non-orthologous proteins, of which 41 cases were from uncultured bacteria and were excluded from our analysis. The remaining 18 instances were found to be good candidates for HGT from different organisms with strong supported bootstrap values.

Of these 18 cases, we detected two instances of HGT that had been previously reported. The transfer of gi:67458540 from *Yersinia* species of gammaproteobacteria to *Rickettsia felis* URRWX Cal2 was reported by Merhej et al., 2011 [26], and the transfer of gi:71084007 from the *CyanobacteriaSynechococcus* and *Prochlorococcus* to two *Candidatus* Pelagibacter ubique strains was reported by Viklund et al., 2011 [22] (Additional file 1, Table S9). Of the remaining 16 cases, 50% of the transfers were observed in *Candidatus* Pelagibacter ubique, and the remaining were present in *Wolbachia* and *Rickettsia* species (Additional file 1, Table S9). These proteins were acquired from different origins, including bacterial species from the *Firmicutes*, *Chlorobi*, *Cyanobacteria*, *Bacteroidetes*, *Actinobacteria* and *Proteobacteria* clades (Epsilon, Delta, Beta and Gamma) as well as from Archaea and Metazoa (*Hydra magnipapillata*). Most of the transferred genes encoded unnamed proteins for which a function has not yet been determined, while others were involved in translocation and hydrolase and synthase activities. To determine the possible origins of these unnamed proteins, we compared them against the ACLAME database; however we were unable to identify genes with similarity above our cutoff value.

### Functional annotation for putative HGT cases (without phylogenetic support) by Gene Ontology (GO)

Out of a total of 1,518 proteins from two groups (838 proteins having at least 1 gain event and 680 proteins from the non-orthologous group), 1,476 remained with no functional annotation. This group was therefore analyzed using the the GO ontology database. No GO terms were identified for 190 of the 1,476 proteins, while the remaining 1,286 proteins were assigned a GO term related to biological processes, molecular functions or cellular components. The results obtained from the GO analysis are described in Additional file 2, Figure S8 and Additional file 1, Table S10. We focused our analysis on the 9th and the 12th DAG levels of the GO hierarchy. At the 9th level, 25 proteins were associated with molecular functions. Of these, 5 were associated with DNA helicase activity and 4 were assigned ATP-dependent helicase activity. The remaining are showed in Additional file 1, Table S11. At the 12th level of the GO hierarchy, 33 proteins were associated with biological process, of which 18 were ATP catabolic and 4 were GTP catabolic. An analysis of the remaining 11 is provided in Additional file 1, Table S12. Thus, only 58 of 1,476 proteins (3.9%) demonstrated significant specificity for a particular GO term.

## Discussion

Although previous studies have detected instances of horizontal gene transfer in *Rickettsiae* species [27-29]. HGTs have not been reported in other genomes such as *Wolbachia*, *Anaplasma* and *Candidatus* Pelagibacter ubique. In the current study, we carried out an exhaustive analysis to investigate additional instances of HGT in 31 complete *Rickettsiales* genomes and detected 38 new instances of HGT as well as 4 cases identified in previously published studies. Among these, several new cases were identified in *Anaplasma* and *Candidatus* Pelagibacter ubique as well as in *Rickettsia* species. Addionally, we carried out a functional analysis of the 42 HGTs identified using our automated pipeline (Figure 1). We considered the remaining cases (1,476 cases) with no phylogenetic support to be putative instances of transfer, and these were analyzed further by searching for GO terms associated with their potential functions.

### HGTs between species from the same environment

We analyzed 42 instances of HGT with phylogenetic support and identified new instances of transfer into the genome of *R. bellii* from different bacterial phyla, including *Firmicutes*, *Nitrospirales*, *Bacteroidetes*, and *Proteobacteria* (Beta and Gamma). *R. bellii* has been previously reported to exchange genes related to amoebal symbionts [26], and there have been no reported cases of gene loss [27]. The newly introduced genes may be maintained as a consequence of the large size of the *R. bellii* genome. Our phylogenetic analyses also identified four instances of horizontal gene transfer among amoeba-resistant microorganisms (Additional file 1, Table S7, gi:157964915, gi:157825906, gi:157827021, gi:229586611). Three out of these four instances were among *Amoebophilus asiasticus* 5a2 and *Rickettsia* species, and one case was among *Legionella* species and *Rickettsia* species. Recently, genes were found to have been transferred between *M. avium* and *L. pneumophila*, which have sympatric lifestyles in free-living protozoa [46]. Additionally, empirical observations of transferred genes have been described in sympatric population [46,47]. Here, our phylogenetic reconstructions identified possible instances of HGT between the ancestors of *Rickettsia* and the amoeba-parasites. Our results support a model in which free-living amoeba act as a “melting pot” for genetic exchange, particularly HGT [47-49]. In addition to amoeba-resistant bacteria, fungi, giant virus and virophages have been detected in free-living protozoa [50-52], which suggests that bacteria living in sympatric populations facilitate more genetic exchange than those living in allopatric populations and that protists can serve as a hot spot for horizontal gene transfer.

We also analyzed instances of HGT from arthropods and their endosymbionts (*Francisella tularensis*). For example, the gene gi:225630595 was transferred from *Francisella tularensis* species to *Wolbachia* endosymbionts of the *Culex* species (Additional file 1, Table S7). This gene encodes for dihydroneopterin aldolase and participates in folate biosynthesis, which is absent from *Wolbachia* species [45]. Other cases include transfers from *Francisella* species to *Orientia* species and to the *Wolbachia* endosymbionts of *Culex quinquefasciatus* Pel (Additional file 1, Table S8, gi:189182975). *Francisella*-like bacterial endosymbionts were discovered in ticks of the *Dermacentor* family [53], and this transfer may have been the result of the co-habitation of *Francisella* and other species within the same host. In this work, we identified an instance of HGT from *Aedes* species to two *Wolbachia* strains (Additional file 1, Table S8, gi:42520378). This transfer was distinct from the case identified by Klasson et al., [44], we identified *Wolbachia* as the recipient species and *Aedes* as the donor species, whereas Klasson et al. reported a transfer in the opposite direction. Our interpretation of this transfer was based on several observations. First, our Bayesian phylogenetic tree for gene gi:4250378 had strong bootstrap support (pp=0.99) (Additional file 2, Figure S7), and the two sequences of *Wolbachia* were affiliated to form a monophyletic group not observed by Klasson et al. Second, we verified our Blast search results for the following 3 sequences: gi:4250378 from the *Wolbachia* endosymbiont of *Drosophila melanogaster*; gi:190571717 from the *Wolbachia* endosymbiont of *Culex quinquefasciatus* Pel; and gi:157104824 from *Aedes aegypti*. Moreover, the two protein sequences from *Wolbachia* demonstrated 62% amino acid identity to each other, but had only 51% amino acid identity to the protein from the *Aedes* species. Third, orthologous genes were not found in any other *Wolbachia* species. Taken together, we believe that this gene transfers occurred from the insect to the bacterium and that this first *Wolbachia* species may have subsequently transferred the gene to other species. Alternatively, the absence of this gene in other *Wolbachia* species may be due to subsequent gene loss. To date, the acquisition of eukaryotic genes by bacteria has rarely been documented. However, a typically eukaryotic glycoside-hydrolase that is necessary for starch breakdown in plants was recently found to have been transferred to bacteria via two successive transfers [54]. Moreover, observations supporting large-scale HGT from bacteria, fungi and plants into *Bdelloid* rotifers have been reported [55]. Horizontal gene transfers between distantly related organisms, i.e., bacteria and multicellular eukaryotes was thought to occur rarely, but our results demonstrate that these transfers may occur more frequently if they are between species that share the same arthropod host, i.e., ticks or beetles. The evolutionary history of these transfers remains a mystery and required further study. Here, we demonstrated two instances of HGT from the *Wolbachia* phage to the *Wolbachia* genome (Additional file 1, Table S8 gi:190570783, Table S9 gi:225630935). *Wolbachia* species habor virus-like particles of the phage WO [56,57], and in cases of Wolbachia-phage transfer, the phage is considered to be the vehicle of transfer via transduction mechanisms rather than the donor species [58].

In addition, we observed many instances of HGT in *Candidatus* Pelagibacter ubique. For example, the gene gi:71083892, which is involved in translocation activity, was transferred from several different species of gammaproteobacteria, such as *Shewanella*, *Aeromonas*, *Vibrio*, *Moritella*, and *Photobacterium* strains, as well as from *Arcobacter butzleri* RM4018 of the epsilonproteobacteria. *Shewanella* species are ubiquitous and are present on the North Atlantic coast as well as deep in the Baltic Sea [59]. Together with *Shewanella* species, *Vibrio* species occupy various aquatic habitats ranging from the deep sea to shallow coastal marine environments [60]. A phylogenetic tree based on the complete sequences of 16S rDNA demonstrated that *Shewanella* species are closely related to marine gammaproteobacteria such as *Aeromonas*, *Vibrio*, *Photobacterium*, *Pseudoalteromonas*, *Colwellia* and *Psychromonas* species [59]. Like *Candidatus* Pelagibacter ubique, these latter bacteria are marine organisms. Evidence of HGT has been reported recently in *Shewanella* species in warm aquatic environments [61]. We also analyzed other cases of HGT in *Candidatus* Nitrospira defluvii (*Nitrospirales*) (Additional file 1, Table S8, gi:157826324) and *Hydra magnipapillata* (Additional file 1, Table S9, gi:71083916) in aquatic environments, although the mechanism responsible for these transfers is not clearly understood. It has been shown that HGTs mediated by gene transfer agents frequently found in marine ecosystems facilitate the adaptation of these microorganisms to new environments [62]. Moreover, mobile genetic elements (MGEs) have been shown to play a crucial role in the expansion of the environmental niches of pathogenic and environmental *Vibrio* species [63]. Taken together, we believe that the aquatic environment may play a crucial role in facilitating such transfers in these organisms.

**Figure 2:**
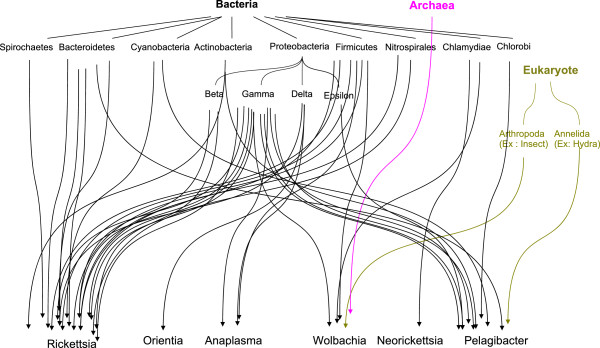
**Rhizome of Rickettsiales genomes.** Bacteria (in black), Archaea (in pink) and Eukaryotes (in yellow).

Overall, we analyzed a number of instances of HGT from different niches, including free-living protozoa, arthropod hosts and aquatic environments. In addition to the characteristic transfers between different ecological niches, HGTs were also analyzed from other origins, including the transfers of genes involved in IS5 family transposase activity from Archaeal species to *Wolbachia* species(Additional file 1, Table S9, gi:42520763). The acquisition of this gene may have been the result of massive gene exchange occurring between bacterial and archaeal species [64]. This horizontal gene transfer from different origins revealed a rhizome for *Rickettsiales* genomes [65] (Figure 2). We also analyzed the functions of novel genes, the origins of which remaine unknown, although previous studies have suggested that these new genes may stem from plasmidic or viral origin [66].

### Functional assignment for putative transfers

Here, we analyzed putative instances of transfer (i.e., those with no phylogenetic support). Only 3.9% of a total of 1,476 proteins were assigned specific terms from the gene ontology database. The majority (18 cases) were assigned terms related to ATP catabolic processes, which are used in nucleotide metabolism, and genes involved in the synthesis for nucleotides are completely lacking in *Rickettsia* species [67]. Moreover, these translocase enzymes were identified in *Rickettsia* species more closely related to amoeba-symbionts than other *Rickettsia*[68-70]. The deficiency of these genes is likely to be linked to the activity of ATP/ADP translocase (*tlc*) which is used to import ATP from the host [71]. This parasitic enzyme may have been duplicated in the *Chlamydiales* ancestor, which may have been transferred to plant and *Rickettsiae* species approximately 1.16 billion years ago. Plant and algae plastids acquired *tlc* at approximately the same time as *Parachlamydiaceae* and *Chlamydiaceae* diverged [72], which suggests that such a transfer may have occurred at a time when *Rickettsiales* and *Chlamydiaceae* shared a common protist ancestral host. Furthermore, these bacteria may have been facultative intracellular bacteria that shared a common ancestral cell host, such as a free-living amoeba [72]. Likewise, it is likely that an ancestor of *Rickettsia* infected an amoeba-like protozoan ancestor and thereby exchanged genes with other symbionts [11]. Furthermore, it has been shown recently that *R. bellii* is able to survive within phagocytic amoeba and can multiply in the nucleus of eukaryotic cells [28]. It has also been reported that *Rickettsiae* species are deficient in *tlc*, which is a parasite enzyme involved in energy metabolism [72]. *Rickettsiae* acquired *tlc* from the *Rickettsiales* ancestor and are therefore able to import ATP from the host cell, whereas members of *Anaplasmataceae* and *Orientia* subsequently lost this gene [72] (Additional file 2, Figure S9). Lacking strong evidence in support of this loss, we therefore can only predict that these proteins may be the result of putative HGT.

## Conclusion

We developed an automated approach for the detection of horizontal gene transfer events that depends on the use of stringent filters. This approach consists of a number of specialized features, including the application of a parsimony method for inferring phyletic patterns followed by a blast filter, automated phylogenetic reconstruction and horizontal gene transfer detection. Using this approach, we identified many new instances of transfer into genomes, including those of *Wolbachia* species, *Anaplasma* species, *Candidatus* Pelagibacter ubique and other species of *Rickettsia*. The current study provides a systematic pipeline for the automated detection of horizontal gene transfer events from several complete proteomic sequences and therefore can be applied to detect the instances of gens transfer for other genomes of interest. This study also provides i) a global analysis of HGT cases in different ecological niches, including protist and arthropods hosts and aquatic environments, and from diverse origins, such as prokaryotic, eukaryotic, archaeal and viral domains, and ii) gene functions resulting from putative instances of gene transfer. Our identification of HGT cases indicative of transfer from different origins allowed us to develop a rhizome for *Rickettsiales* genomes.

## Methods

### Complete Proteomes

The protein sets for 31 completely sequenced species (13 *Rickettsia* strains, 2 *Orientia* strains, 4 *Anaplasma* strains, 4 *Ehrlichia* strains, 4 *Wolbachia* strains, 2 *Neorickettsia* strains, *Candidatus* Pelagibacter ubique HTCC1602 and *Caulobacter crescentus* CB15) within the *Rickettsiales* order were downloaded from NCBI (ftp://ftp.ncbi.nih.gov/genomes/Bacteria/). The details regarding the NCBI accession numbers for these genomes are provided in Additional file 1, Table S1.

### Detection of orthologous groups

We employed OrthoMCL [73] to retrieve orthologous proteins. Only protein sequences longer than 50 amino acid residues were considered for further analysis. Homologous sequences were selected using the all-against-all BlastP algorithm [74] with an E value of less than 0.00001. Then, clustering of the orthologous sequences was analyzed using the Markov Cluster algorithm [75] which was based on probability and graph flow theory that allows the simultaneous classification of global relationships in a similarity space. The inflation index of 1.5 was used to regulate cluster tightness (granularity), and the resulting clustered ortholog groups were analyzed further.

### 16S rRNA tree for Rickettsiales

We reconstructed a phylogenetic tree using 16S rRNA sequences to form a species tree. The 16S RNA sequences were downloaded from NCBI (ftp://ftp.ncbi.nih.gov/genomes/Bacteria/), and the alignments were carried out using MUSCLE 3.6 [76]. Next, the phylogenetic tree was constructed with Mega5 software [77] and the following three methods: Neighbor Joining (NJ), Maximum Parsimony(MP) and Maximum Likelihood (ML). The 16S rRNA species tree is provided as Additional file 2, Figure S1.

### Detection of gene gain and loss by phyletic pattern

The ortholog groups identified by OrthoMCL were submitted to PhyloPattern [78], a software library based on the Prolog language [79], for the automated analysis and manipulation of phylogenetic trees (within the DAGOBAH framework). PhyloPattern contains three modules: (1) a tree annotation module that incorporates a traversal algorithm, infers phylogenetic trees composed of hierarchical nodes as binary structures, and functionally annotates each node; (2) a pattern-matching module to define patterns that can be used to search phylogenetic trees; and (3) a tree comparison module for the global comparison of tree topologies and the identification of matching nodes.

The 16S rRNA species tree was used as a reference for inferring topologies for further analysis. We mapped the characteristics “presence” and “absence” for genes on the nodes of the species tree using the tree annotation module. The presence and absence characteristics corresponded to the presence and absence of the gene in a species from a group of orthologs at a particular node. Next, we defined the distribution of ancestral characteristics by implementing the Sankoff parsimony algorithm [80]. Further analyses were carried out to search for gene gain and loss events in phylogenetic subtrees using the pattern-matching module in PhyloPattern [61]. A phylogenetic subtree consisted of an ancestral node and two descendant nodes. The pattern was defined as a loss event if the gene was present in the ancestral node and one descendant node but absent in another node. Conversely, the pattern wass defined as a gain event if the gene was absent in the ancestral node and one descendant node but was present in another descendant node (Additional file 2, Figure S2). This pattern for each local subtree was then used with the tree comparison module to infer the topologies of global trees. The total number of gain and loss events was subsequently calculated as the sum of all events of the subtrees. The gain and loss events inferred from the PhyloPattern are shown in Additional file 2, Figure S2 and Figure S3. Finally, one representative protein sequence from each ortholog group was analyzed using the Blast program. The selection of sequences was carried out in the following order: *Rickettsia* species, *Orientia* species, *Wolbachia* species, *Anaplasma* species, *Ehrlichia* species, *Neorickettsia* species and *Candidatus* Pelagibacter ubique.

### Analysis of homologous sequences using the Blast filter

Based on the number of gene gain and loss events identified using PhyloPattern, one protein sequence from each representative event was analyzed by Blast search. We speculated that gain events may have been the result of HGT and that loss events may have been the result of gene loss during the course of a species’ evolution. As the main objective of the current study was to analyze HGT, we evaluated only instances of gene gain unless otherwise stated.

We carried out the BlastP search using cutoff E-values less than 10-10 against the NR NCBI database using the remote method. The first hundred homologous protein sequences from each query were retrieved and classified into two groups: (a). hits within the group of interest (within alphaproteobacteria) and (b). hits outside of the group of interest (out of the alphaproteobacteria). The protein sequences outside of the group of interest were retrieved and subjected to further phylogenetic analysis. We chose only those Blast results that returned at least one hit outside of alphaproteobacteria for further analysis. Because the results of the Blast search that provided hits only within the alphaproteobacteria were not considered for further analysis, these cases were not analyzed in the phylogenetic reconstructions. Interestingly, we also detected several protein sequences in the studied group that were lineage-specific, and we presumed that these represented new genes or novel genes. The origin of new genes can result from many types of genetic events, such as gene duplication, gene fission and fusion, lateral gene transfer, transposable elements (TEs) and de novo gene formation [81].

### Phylogeny construction

The results obtained from the Blast analysis were submitted to FIGENIX [82,83] for phylogenetic reconstruction within the DAGOBAH framework. FIGENIX is an automated pipeline for structural and functional annotation in which the structural part represents the integration of abinitio and homology-based approaches and the functional annotation consists of fully automated modules for complex phylogenomic inference. Homologous sequences were retrieved from the NR NCBI database by BlastP search and were aligned using MUSCLE v3.6 [59]. Functional domains were detected for all aligned sequences using the HMMPFAM [84] database. Ambiguously aligned regions, including data that produced bias or noise (such as short sequences), were automatically excluded from the analysis, and a new alignment was built by merging the preserved parts of the domain alignments. Based on the new alignment, phylogenetic trees were constructed using three different methods: NJ with ClustalW software [85], MP using the PAUP package [86] (p-distance) and ML using the TREE-PUZZLE package with the default parameters [87]. The topologies of these trees were compared using the PSCORE command (“Templeton winning sites” test) from the PAUP package and the KISHINO-HASEGAWA test [88] from the TREE-PUZZLE package. All three trees were then merged into a final consensus tree. The consensus tree was compared to a reference species tree (Tree of Life) from NCBI, and phylogenies were inferred for orthologous protein sequences against the query sequence. The topology of the 16S rRNA sequence and the tree from NCBI were similar with the exception that the NCBI tree included nodes that were not bifurcated.

### Detection of gene transfer events by HGT agent

The output generated by FIGENIX was submitted to the multi-agent system DAGOBAH [89], in which HGT events were detected using an in-house-built transfer filter called HGT agent. HGT agent uses PhyloPattern [61] to annotate each internal duplication node of the tree with three tags, including: recipient species, donor species and external species. Then, it applies a special phyletic pattern and searches the gene tree to find recipient species that are closer to donor species than to other external species that would otherwise be placed between the recipient and donor species in the species tree. In other words, a “donor” subtree must contain only species of a specific group and not those from the “recipient” group and vice versa, and there should be no common species between the donor and external groups. Using this agent, one can specify the names of the donor species and the recipient species according to their usage. In the current study, we defined the recipient species as the *Rickettsiales* group, the donor species as the non-alphaproteobacterial species and external species as different than donor species. We considered the donor species to be the most likely donors. The pattern used to detect HGT is shown in Additional file 2, Figure S4. In this way, the HGT agent used phyletic patterns to detect instances of HGT with strongly supported bootstrap values (greater than 50%), which were than confirmed by Bayesian methods to validate the results. The results of the Bayesian analysis are presented in Additional file 1, Table S2 and Additional file 2, Figure S7.

### Confirmation of HGT events by Bayesian analysis

We used Phylobayes 3.2 [90] for the Bayesian analysis to detect HGT cases. Phylobayes was run using the CAT model and a gamma correction with 4 rate categories [91]. Four chains were run for at least two million generations. The first 250 trees were discarded as “burn-in”, and the remaining trees from each chain were used to test for convergence (less than 0.1) and computed for consensus trees. The trees were visualized using FigTree 3.1, a program for the graphical viewing of phylogenetic trees (http://tree.bio.ed.ac.uk/software/figtree/).

### Detection of transfer from mobile genetic elements using the ACLAME database

To determine the possible origins of the hypothetical transferred proteins, we verified these against the ACLAME database [92], which is a collection of known mobile genetic elements (MGEs) from phage genomes, plasmids and transposons. BlastP searches were performed for sequences with an E-value of less than 10-3 and a percent of identity greater than 50%.

### Functional annotation by Gene Ontology

Gene ontology can be represented as a direct acyclic graph (DAG) with a hierarchical structure encompassing numerous levels and terms (Gene Ontology project 2006) [93]. Genes associated with GO terms are more likely to be functionally enriched at each DAG level. General biological roles are identified at the higher levels, and specific roles are identified at the lower levels. Deeper DAG levels indicate high specificity for the gene [94]. As functions for the 42 HGT cases had been previously discussed, they were not included in the GO analysis. Best hit BlastP (E-values less than 0.0001) for each of the 1,476 proteins against the GO database were retrieved and analyzed.

### Data Access

For cases of horizontal gene transfer, the Genbank IDs for the phylogenetic trees resulting from at least 1 gain and 1 loss event and the non-orthologous proteins are stored and can be obtained from the I.O.D.A. database [95] (http://ioda.univ-provence.fr).

## Abbreviations

HGT: Horizontal gene transfer; NR: Non-redundant; COG: Cluster of Orthologous Groups.

## Competing interests

The authors declare that they have no competing interests.

## Authors’ contributions

PP and DR conceived of and designed the study. PG supervised the software development. LG and PTL developed the bioinformatic tool to adapt DAGOBAH to bacteria and the ortholog filter for the OrthoMCL groups. JP developed a preliminary version of the Blast filter, the HGT detection agent and the graphical interface package that visualized the results deployed on the I.O.D.A web site. OC installed OrthoMCL and performed the GO annotation. PTL performed the data production and the analysis. PTL and HGR analyzed the phylogenies. PTL, HGR, PP and DR wrote the manuscript. PTL, HGR, DR and PP analyzed the data. All authors read and approved the final manuscript.

## Supplementary Material

Additional file 1 **Table S1 shows the NCBI accession numbers for the studied species.Table S2** shows the Bayesian analysis for the horizontal gene transfer cases from other species to Rickettsiales excluding alphaproteobacteria. Violet rows represent transfer cases from at least 1 gain event, and the brown rows represent transfer cases from a non-orthologous group. PP indicates the posterior probability value from the Bayesian analysis, aa indicates amino acids, and BV indicates the bootstrap values obtained with the NJ, MP, and ML methods. **Table S3** is a list of annotations for the conserved genes from 267 Ortholog clusters of *Rickettsiales* and *Caulobacter cresentus* CB15 from the COG database. **Table S4** is a list of annotations for the conserved genes from 277 Ortholog clusters of *Rickettsia*, *Orientia*, *Wolbachia*, *Anaplasma*, *Ehrlichia*, *Neorickettsia* and *Candidatus* Pelagibacter ubique from the COG database. **Table S5** is a list of annotation for the conserved genes from 328 Ortholog clusters of *Rickettsia*, *Orientia*, *Wolbachia*, *Anaplasma*, *Ehrlichia* and *Neorickettsia* from the COG database. **Table S6** is a list of annotation sfor the 1,709 orthologous groups based on the COG database. **Table S7** is a table of the horizontal gene transfer cases identified from at least 1 gain and 1 loss event (part 1). **Table S8** is a table of the horizontal gene transfer cases identified from at least 1 gain and 1 loss event (part 2). **Table S9** is a table of the horizontal gene transfer cases identified from non-orthologous groups. **Table S10** is a table showing the distribution of proteins annotated from the Gene Ontology database. **Table S11** is a table showing the assignment of proteins annotated by Gene Ontology at the 9th level. **Table S12** is a table showing the assignment of proteins annotated by Gene Ontology at the 12th level. **Table S13** is a table showing the non-synonymous/synonymous substitution analysis for de novo genes. Click here for file

Additional file 2 **Figure S1 shows the 16S rRNA species tree.Figure S2** shows the two principle patterns for the detection of gene gain and gene loss using PhyloPattern: a. Loss event and b. Gain event. Red circles represent the “present” characteristic, blue circles represent the “absent” characteristic. The pattern was defined as a loss event if the gene was present in the ancestral node and one descendant node but absent in another node (violet rhomboid). Conversely, the pattern was defined as a gain event if the gene was absent in the ancestral node and one descendant node but was present in another descendant node (green rhomboid). **Figure S3** maps the gain and loss events from the phyletic patterns onto the species tree. Red circles represent the “present” characteristic, and blue circles represent the “absent” characteristic. Violet and green rhomboids represent loss and gain events, respectively. **Figure S4** shows the transfer pattern detected by HGT agent from the multi-agent system DAGOBAH. HGT agent applied PhyloPattern to annotate the subtree (see Methods). Yellow circle with a D indicates that a duplication of the gene occurred at that node. Red, green and cyan circles represent recipient species, donor species and external species, respectively. Tax id: taxonomy identity. **Figure S5** shows the results of the number of orthologous groups obtained from the gene gain and gene loss analysis by PhyloPattern in each respective event.
**Figure S6a** is a flowchart representing the detailed description of the systematic analysis of horizontal gene transfer detection with at least one gain event. **Figure S6b** is a flowchart representing the detailed description of the systematic analysis of horizontal gene transfer detection for non-orthologous groups. **Figure S7** shows Bayesian phylogenetic trees for a subset of the detected instances of horizontal gene transfer from the group with at least 1 gain and 1 loss (No. 1 to 10) and the non-orthologous groups (No. 11 to 15). The scale bar represents the average number of substitutions per site. Numbers at nodes correspond to the posterior probabilities calculated by Phylobayes. The donor species are indicated in blue, and the recipient species are indicated in red. **Figure S8** represents the distribution of proteins assignments using the Gene Ontology database. **Figure S9** illustrates the hypothesis for the acquisition of the ADP/ATP translocase-encoding gene by *Rickettsia* species. The evolutionary time was estimated from the data provided by Greub and Raoult, 2003. **Figure S10** is a histogram of COG size. Click here for file
