# Supplementary material for: An automated approach for the identification of horizontal gene transfers from complete genomes reveals the rhizome of Rickettsiales
Source: BMC Evol Biol. 2012 Dec 12;12:243. doi: 10.1186/1471-2148-12-243 (PMC3575314; doi:10.1186/1471-2148-12-243)
Supplement: Additional file 2 — Figure S1 shows the 16S rRNA species tree.Figure S2 shows the two principle patterns for the detection of gene gain and gene loss using PhyloPattern: a. Loss event and b. Gain event. Red circles represent the “present” characteristic, blue circles represent the “absent” characteristic. The pattern was defined as a loss event if the gene was present in the ancestral node and one descendant node but absent in another node (violet rhomboid). Conversely, the pattern was defined as a gain event if the gene was absent in the ancestral node and one descendant node but was present in another descendant node (green rhomboid). Figure S3 maps the gain and loss events from the phyletic patterns onto the species tree. Red circles represent the “present” characteristic, and blue circles represent the “absent” characteristic. Violet and green rhomboids represent loss and gain events, respectively. Figure S4 shows the transfer pattern detected by HGT agent from the multi-agent system DAGOBAH. HGT agent applied PhyloPattern to annotate the subtree (see Methods). Yellow circle with a D indicates that a duplication of the gene occurred at that node. Red, green and cyan circles represent recipient species, donor species and external species, respectively. Tax id: taxonomy identity. Figure S5 shows the results of the number of orthologous groups obtained from the gene gain and gene loss analysis by PhyloPattern in each respective event. Figure S6a is a flowchart representing the detailed description of the systematic analysis of horizontal gene transfer detection with at least one gain event. Figure S6b is a flowchart representing the detailed description of the systematic analysis of horizontal gene transfer detection for non-orthologous groups. Figure S7 shows Bayesian phylogenetic trees for a subset of the detected instances of horizontal gene transfer from the group with at least 1 gain and 1 loss (No. 1 to 10) and the non-orthologous groups (No. 11 to 15). The scale bar represents [file 1471-2148-12-243-S2.ZIP › Additional data file 2 revised/Figure S10.pdf]

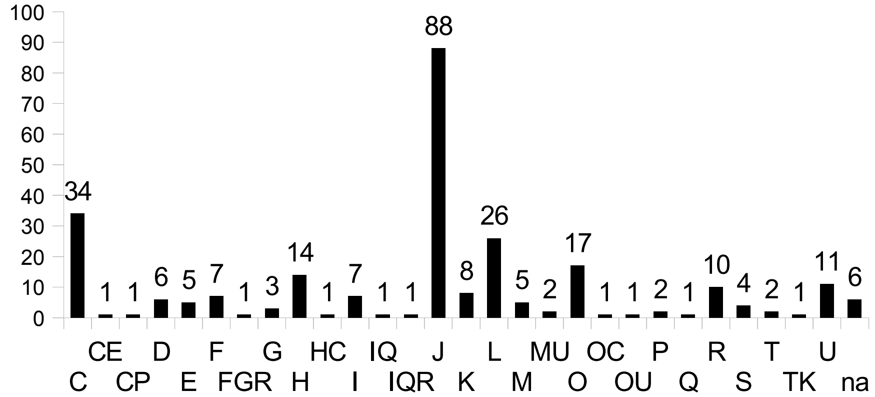

Figure S10: A. Histogram of distribution of 267 COG groups between *Rickettsiales* and *Caulobacter cresentus* CB15 from COG database. J, Translation; A, RNA processing and modification; K, Transcription; L, Replication, recombination and repair; B, Chromatin structure and dynamics; D, Cell cycle control, mitosis and meiosis; Y, Nuclear structure; V, Defense mechanisms; T, Signal transduction mechanisms; M, Cell wall/membrane biogenesis; N, Cell motility; Z, Cytoskeleton

; W, Extracellular structures; U, Intracellular trafficking and secretion; O, Posttranslational modification, protein turnover, chaperones; C, Energy production and conversion; G, Carbohydrate transport and metabolism; E, Amino acid transport and metabolism; F, Nucleotide transport and metabolism; H, Coenzyme transport and metabolism; I, Lipid transport and metabolism; P, Inorganic ion transport and metabolism; Q, Secondary metabolites biosynthesis, transport and catabolism; R, General function prediction only; S, Function unknown; na, Not in COGs

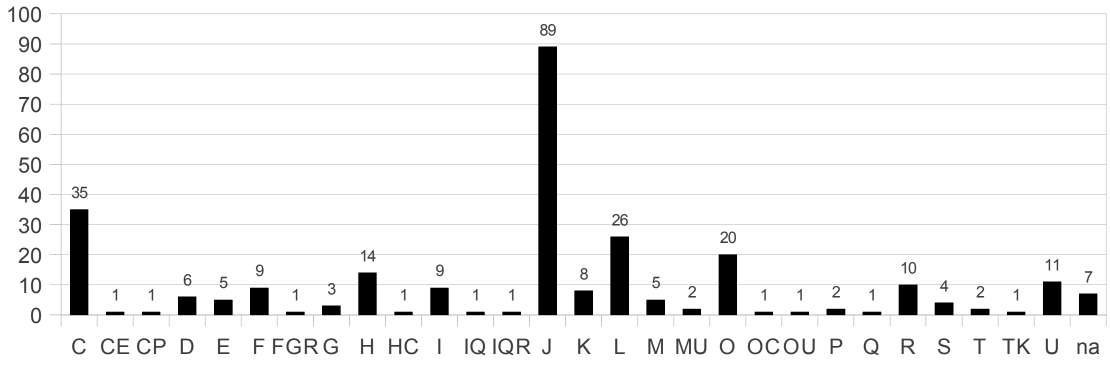

Figure S10: B. Histogram of distribution of 267 COG groups among *Rickettsia, Orientia*, *Wolbachia, Anaplasma, Ehrlichia, Neorickettsia* and *Candidatus* Pelagibacter ubique from COG database. The letters signification were indicated in the figure legend above.

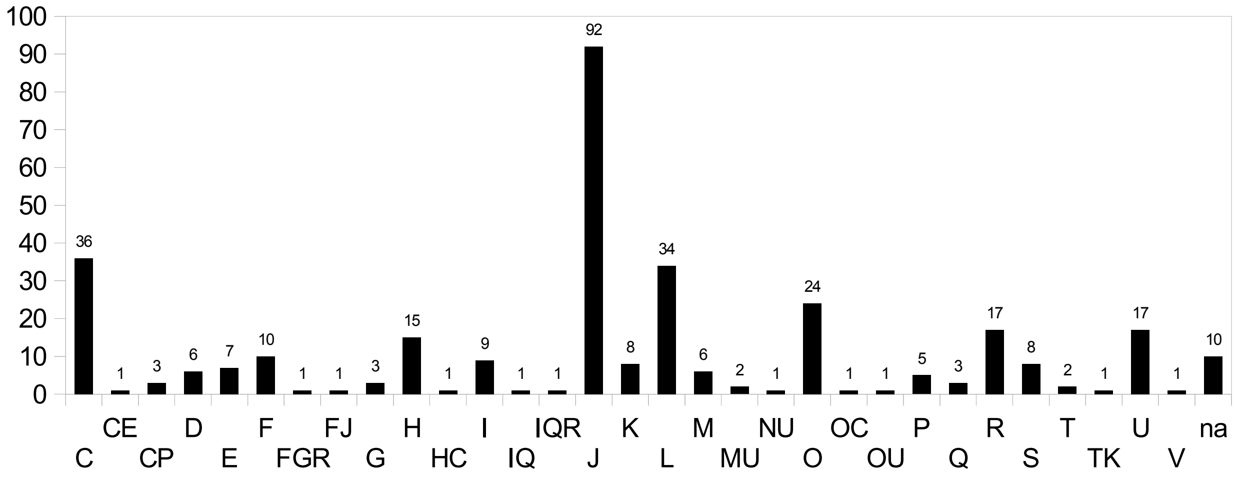

Figure S10: C. Histogram of distribution of 328 COG groups among *Rickettsia, Orientia*, *Wolbachia, Anaplasma, Ehrlichia* and *Neorickettsia* from COG database. The letters signification were indicated in the figure legend above.

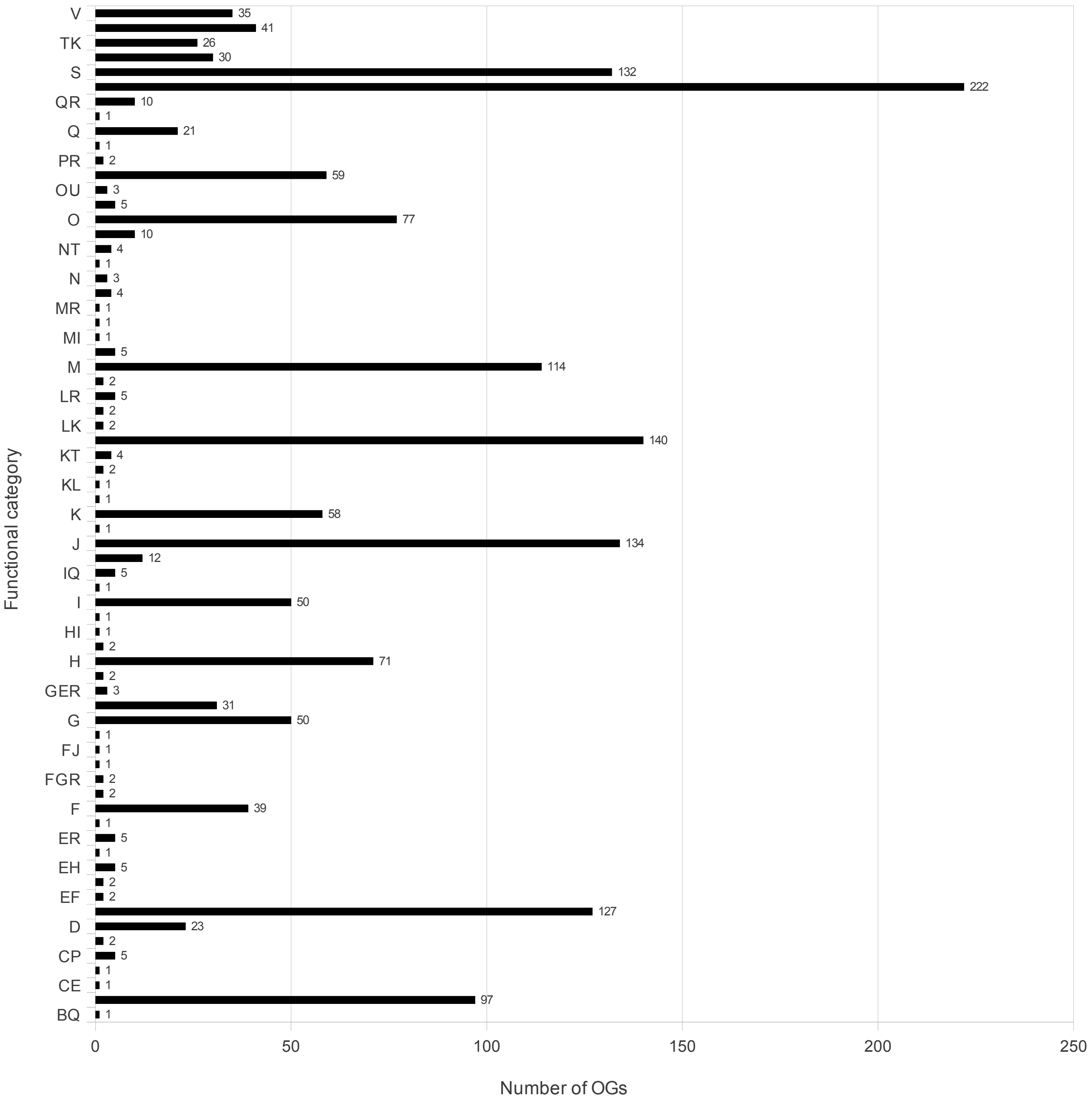

Figure S10: D. histogram of distribution of 1,709 COG groups based on COG database. The letters signification were indicated in figure legend above.
