# Supplementary figures and images for: An automated approach for the identification of horizontal gene transfers from complete genomes reveals the rhizome of Rickettsiales
Source: BMC Evol Biol. 2012 Dec 12;12:243. doi: 10.1186/1471-2148-12-243 (PMC3575314; doi:10.1186/1471-2148-12-243)

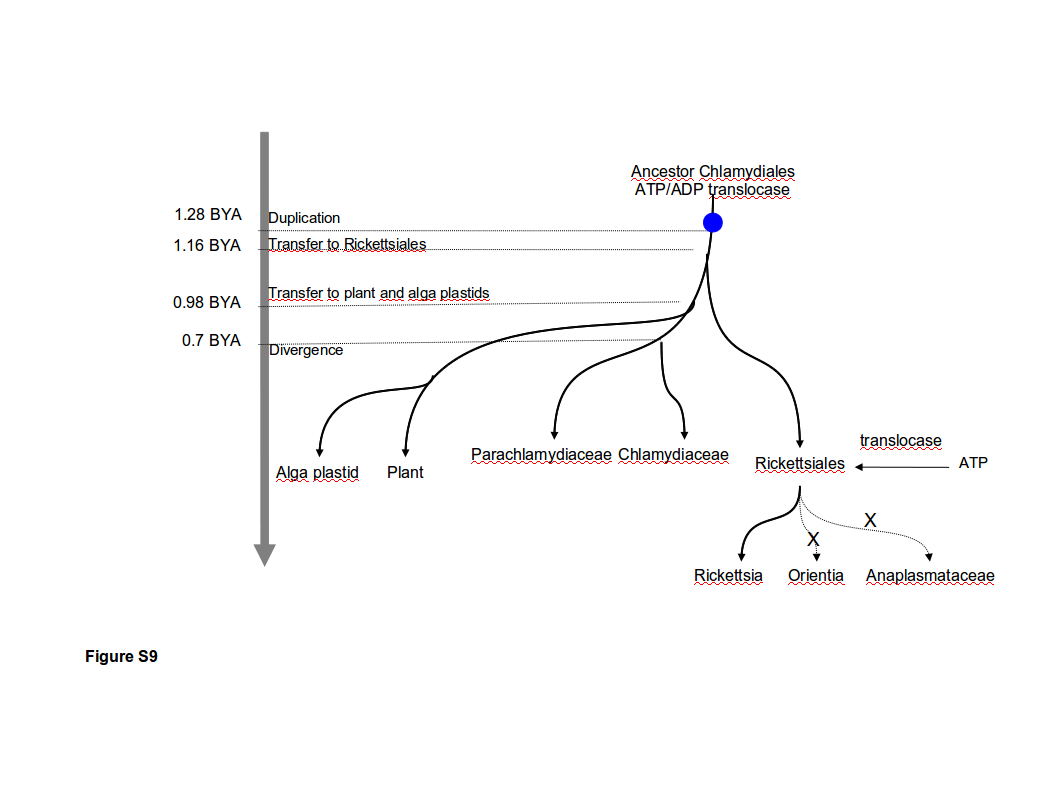

Supplement: Additional file 2 — Figure S1 shows the 16S rRNA species tree.Figure S2 shows the two principle patterns for the detection of gene gain and gene loss using PhyloPattern: a. Loss event and b. Gain event. Red circles represent the “present” characteristic, blue circles represent the “absent” characteristic. The pattern was defined as a loss event if the gene was present in the ancestral node and one descendant node but absent in another node (violet rhomboid). Conversely, the pattern was defined as a gain event if the gene was absent in the ancestral node and one descendant node but was present in another descendant node (green rhomboid). Figure S3 maps the gain and loss events from the phyletic patterns onto the species tree. Red circles represent the “present” characteristic, and blue circles represent the “absent” characteristic. Violet and green rhomboids represent loss and gain events, respectively. Figure S4 shows the transfer pattern detected by HGT agent from the multi-agent system DAGOBAH. HGT agent applied PhyloPattern to annotate the subtree (see Methods). Yellow circle with a D indicates that a duplication of the gene occurred at that node. Red, green and cyan circles represent recipient species, donor species and external species, respectively. Tax id: taxonomy identity. Figure S5 shows the results of the number of orthologous groups obtained from the gene gain and gene loss analysis by PhyloPattern in each respective event. Figure S6a is a flowchart representing the detailed description of the systematic analysis of horizontal gene transfer detection with at least one gain event. Figure S6b is a flowchart representing the detailed description of the systematic analysis of horizontal gene transfer detection for non-orthologous groups. Figure S7 shows Bayesian phylogenetic trees for a subset of the detected instances of horizontal gene transfer from the group with at least 1 gain and 1 loss (No. 1 to 10) and the non-orthologous groups (No. 11 to 15). The scale bar represents [file 1471-2148-12-243-S2.ZIP › Additional data file 2 revised/Figure S9.png]

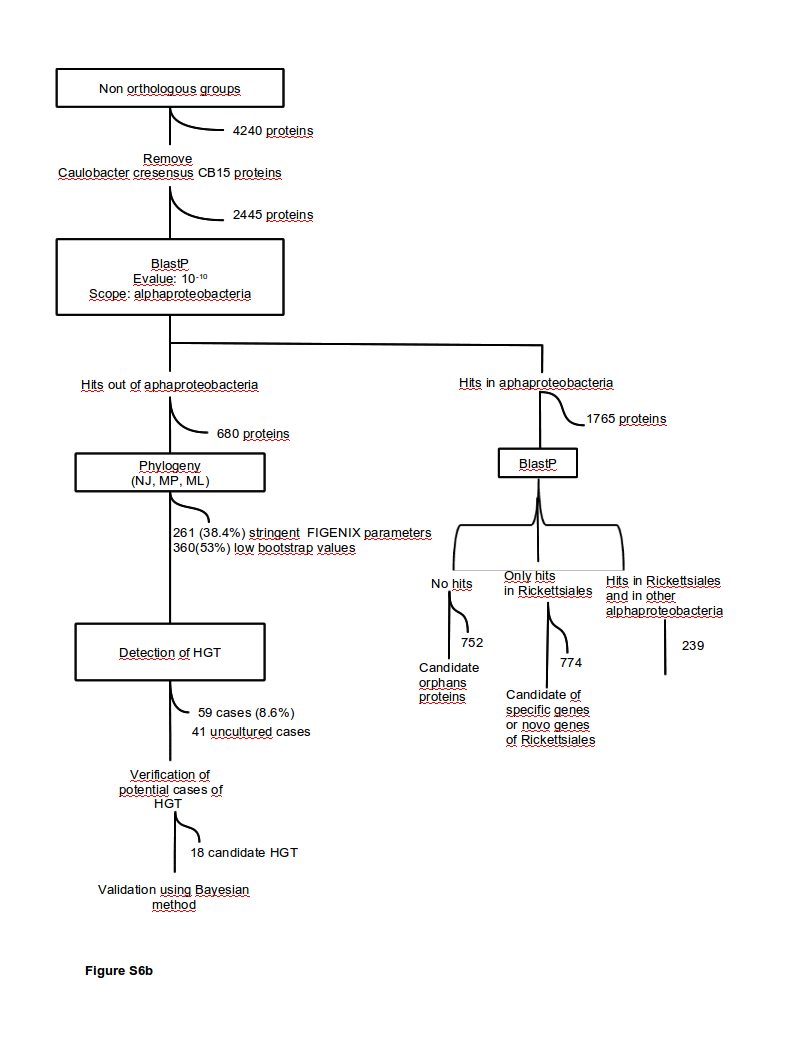

Supplement: Additional file 2 — Figure S1 shows the 16S rRNA species tree.Figure S2 shows the two principle patterns for the detection of gene gain and gene loss using PhyloPattern: a. Loss event and b. Gain event. Red circles represent the “present” characteristic, blue circles represent the “absent” characteristic. The pattern was defined as a loss event if the gene was present in the ancestral node and one descendant node but absent in another node (violet rhomboid). Conversely, the pattern was defined as a gain event if the gene was absent in the ancestral node and one descendant node but was present in another descendant node (green rhomboid). Figure S3 maps the gain and loss events from the phyletic patterns onto the species tree. Red circles represent the “present” characteristic, and blue circles represent the “absent” characteristic. Violet and green rhomboids represent loss and gain events, respectively. Figure S4 shows the transfer pattern detected by HGT agent from the multi-agent system DAGOBAH. HGT agent applied PhyloPattern to annotate the subtree (see Methods). Yellow circle with a D indicates that a duplication of the gene occurred at that node. Red, green and cyan circles represent recipient species, donor species and external species, respectively. Tax id: taxonomy identity. Figure S5 shows the results of the number of orthologous groups obtained from the gene gain and gene loss analysis by PhyloPattern in each respective event. Figure S6a is a flowchart representing the detailed description of the systematic analysis of horizontal gene transfer detection with at least one gain event. Figure S6b is a flowchart representing the detailed description of the systematic analysis of horizontal gene transfer detection for non-orthologous groups. Figure S7 shows Bayesian phylogenetic trees for a subset of the detected instances of horizontal gene transfer from the group with at least 1 gain and 1 loss (No. 1 to 10) and the non-orthologous groups (No. 11 to 15). The scale bar represents [file 1471-2148-12-243-S2.ZIP › Additional data file 2 revised/Figure S6b.png]

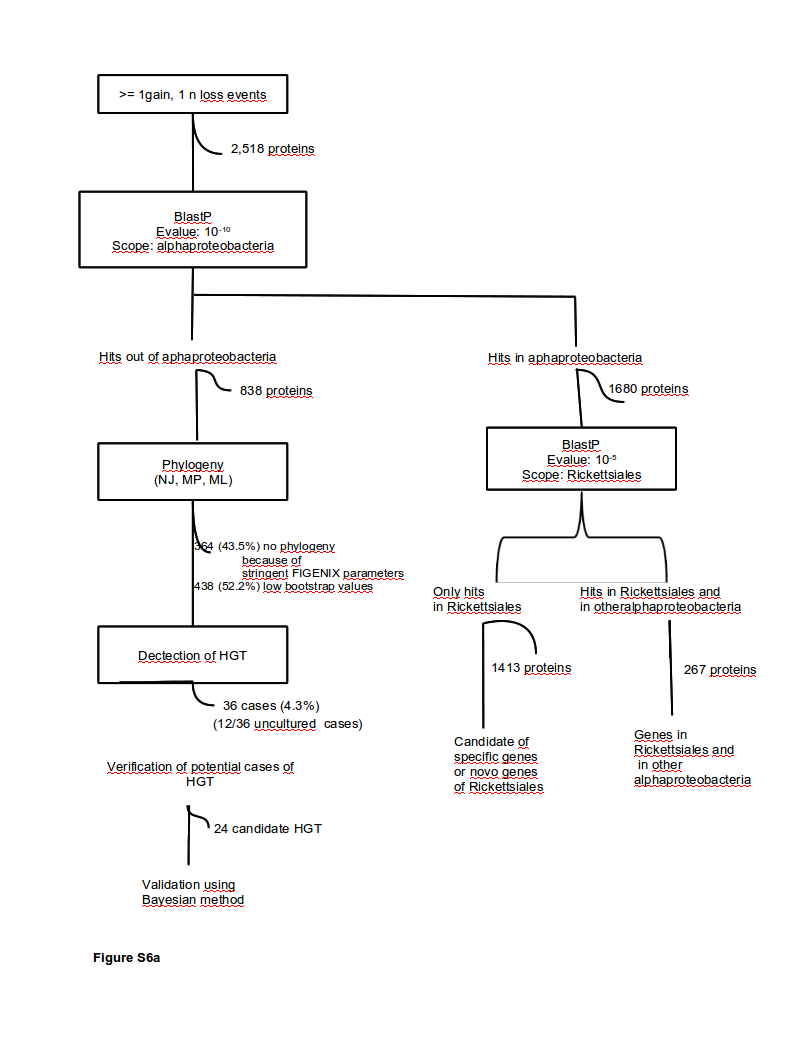

Supplement: Additional file 2 — Figure S1 shows the 16S rRNA species tree.Figure S2 shows the two principle patterns for the detection of gene gain and gene loss using PhyloPattern: a. Loss event and b. Gain event. Red circles represent the “present” characteristic, blue circles represent the “absent” characteristic. The pattern was defined as a loss event if the gene was present in the ancestral node and one descendant node but absent in another node (violet rhomboid). Conversely, the pattern was defined as a gain event if the gene was absent in the ancestral node and one descendant node but was present in another descendant node (green rhomboid). Figure S3 maps the gain and loss events from the phyletic patterns onto the species tree. Red circles represent the “present” characteristic, and blue circles represent the “absent” characteristic. Violet and green rhomboids represent loss and gain events, respectively. Figure S4 shows the transfer pattern detected by HGT agent from the multi-agent system DAGOBAH. HGT agent applied PhyloPattern to annotate the subtree (see Methods). Yellow circle with a D indicates that a duplication of the gene occurred at that node. Red, green and cyan circles represent recipient species, donor species and external species, respectively. Tax id: taxonomy identity. Figure S5 shows the results of the number of orthologous groups obtained from the gene gain and gene loss analysis by PhyloPattern in each respective event. Figure S6a is a flowchart representing the detailed description of the systematic analysis of horizontal gene transfer detection with at least one gain event. Figure S6b is a flowchart representing the detailed description of the systematic analysis of horizontal gene transfer detection for non-orthologous groups. Figure S7 shows Bayesian phylogenetic trees for a subset of the detected instances of horizontal gene transfer from the group with at least 1 gain and 1 loss (No. 1 to 10) and the non-orthologous groups (No. 11 to 15). The scale bar represents [file 1471-2148-12-243-S2.ZIP › Additional data file 2 revised/Figure S6a.png]

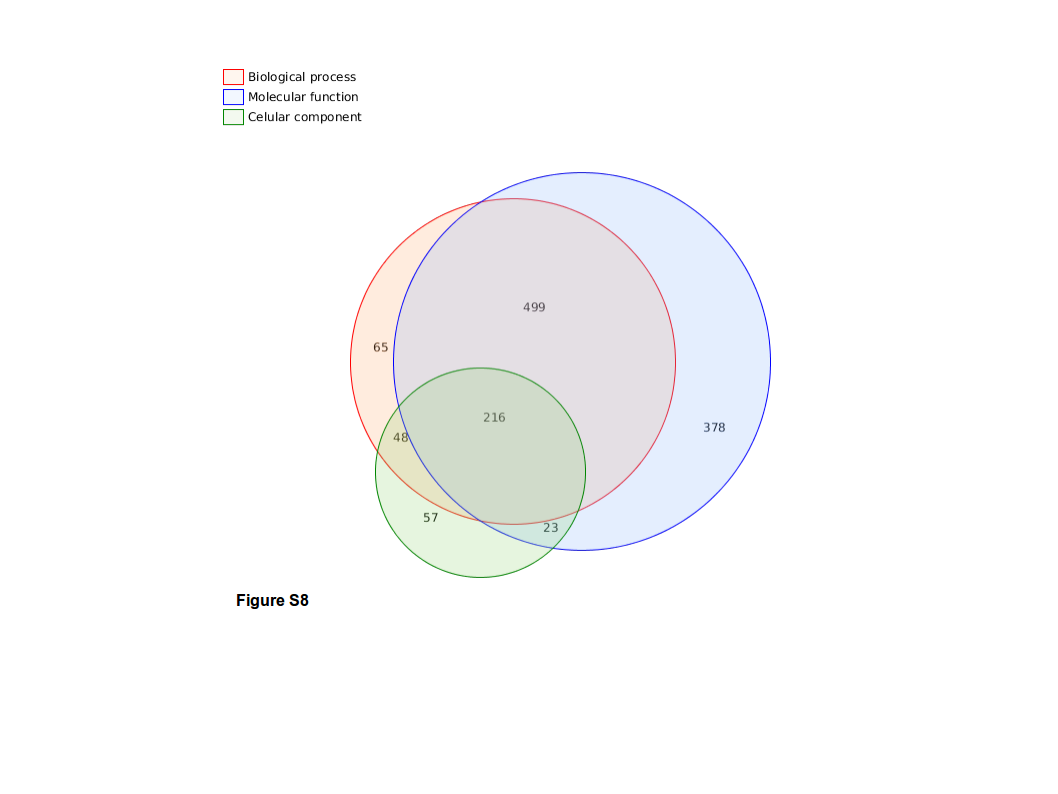

Supplement: Additional file 2 — Figure S1 shows the 16S rRNA species tree.Figure S2 shows the two principle patterns for the detection of gene gain and gene loss using PhyloPattern: a. Loss event and b. Gain event. Red circles represent the “present” characteristic, blue circles represent the “absent” characteristic. The pattern was defined as a loss event if the gene was present in the ancestral node and one descendant node but absent in another node (violet rhomboid). Conversely, the pattern was defined as a gain event if the gene was absent in the ancestral node and one descendant node but was present in another descendant node (green rhomboid). Figure S3 maps the gain and loss events from the phyletic patterns onto the species tree. Red circles represent the “present” characteristic, and blue circles represent the “absent” characteristic. Violet and green rhomboids represent loss and gain events, respectively. Figure S4 shows the transfer pattern detected by HGT agent from the multi-agent system DAGOBAH. HGT agent applied PhyloPattern to annotate the subtree (see Methods). Yellow circle with a D indicates that a duplication of the gene occurred at that node. Red, green and cyan circles represent recipient species, donor species and external species, respectively. Tax id: taxonomy identity. Figure S5 shows the results of the number of orthologous groups obtained from the gene gain and gene loss analysis by PhyloPattern in each respective event. Figure S6a is a flowchart representing the detailed description of the systematic analysis of horizontal gene transfer detection with at least one gain event. Figure S6b is a flowchart representing the detailed description of the systematic analysis of horizontal gene transfer detection for non-orthologous groups. Figure S7 shows Bayesian phylogenetic trees for a subset of the detected instances of horizontal gene transfer from the group with at least 1 gain and 1 loss (No. 1 to 10) and the non-orthologous groups (No. 11 to 15). The scale bar represents [file 1471-2148-12-243-S2.ZIP › Additional data file 2 revised/Figure S8.png]

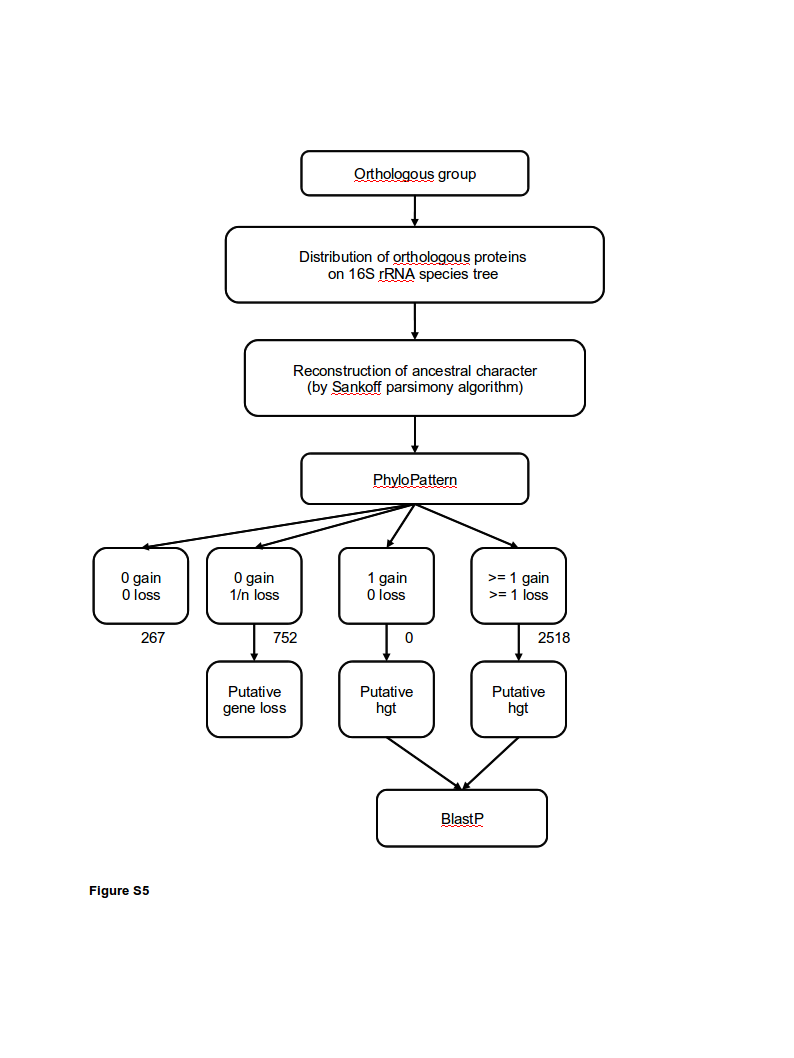

Supplement: Additional file 2 — Figure S1 shows the 16S rRNA species tree.Figure S2 shows the two principle patterns for the detection of gene gain and gene loss using PhyloPattern: a. Loss event and b. Gain event. Red circles represent the “present” characteristic, blue circles represent the “absent” characteristic. The pattern was defined as a loss event if the gene was present in the ancestral node and one descendant node but absent in another node (violet rhomboid). Conversely, the pattern was defined as a gain event if the gene was absent in the ancestral node and one descendant node but was present in another descendant node (green rhomboid). Figure S3 maps the gain and loss events from the phyletic patterns onto the species tree. Red circles represent the “present” characteristic, and blue circles represent the “absent” characteristic. Violet and green rhomboids represent loss and gain events, respectively. Figure S4 shows the transfer pattern detected by HGT agent from the multi-agent system DAGOBAH. HGT agent applied PhyloPattern to annotate the subtree (see Methods). Yellow circle with a D indicates that a duplication of the gene occurred at that node. Red, green and cyan circles represent recipient species, donor species and external species, respectively. Tax id: taxonomy identity. Figure S5 shows the results of the number of orthologous groups obtained from the gene gain and gene loss analysis by PhyloPattern in each respective event. Figure S6a is a flowchart representing the detailed description of the systematic analysis of horizontal gene transfer detection with at least one gain event. Figure S6b is a flowchart representing the detailed description of the systematic analysis of horizontal gene transfer detection for non-orthologous groups. Figure S7 shows Bayesian phylogenetic trees for a subset of the detected instances of horizontal gene transfer from the group with at least 1 gain and 1 loss (No. 1 to 10) and the non-orthologous groups (No. 11 to 15). The scale bar represents [file 1471-2148-12-243-S2.ZIP › Additional data file 2 revised/Figure S5.png]

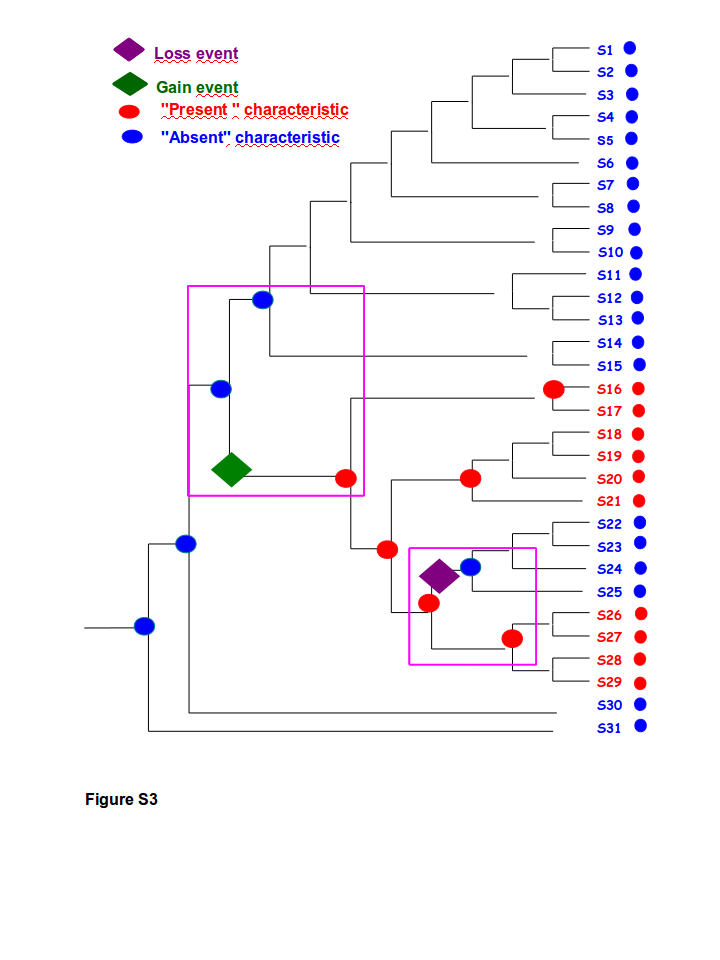

Supplement: Additional file 2 — Figure S1 shows the 16S rRNA species tree.Figure S2 shows the two principle patterns for the detection of gene gain and gene loss using PhyloPattern: a. Loss event and b. Gain event. Red circles represent the “present” characteristic, blue circles represent the “absent” characteristic. The pattern was defined as a loss event if the gene was present in the ancestral node and one descendant node but absent in another node (violet rhomboid). Conversely, the pattern was defined as a gain event if the gene was absent in the ancestral node and one descendant node but was present in another descendant node (green rhomboid). Figure S3 maps the gain and loss events from the phyletic patterns onto the species tree. Red circles represent the “present” characteristic, and blue circles represent the “absent” characteristic. Violet and green rhomboids represent loss and gain events, respectively. Figure S4 shows the transfer pattern detected by HGT agent from the multi-agent system DAGOBAH. HGT agent applied PhyloPattern to annotate the subtree (see Methods). Yellow circle with a D indicates that a duplication of the gene occurred at that node. Red, green and cyan circles represent recipient species, donor species and external species, respectively. Tax id: taxonomy identity. Figure S5 shows the results of the number of orthologous groups obtained from the gene gain and gene loss analysis by PhyloPattern in each respective event. Figure S6a is a flowchart representing the detailed description of the systematic analysis of horizontal gene transfer detection with at least one gain event. Figure S6b is a flowchart representing the detailed description of the systematic analysis of horizontal gene transfer detection for non-orthologous groups. Figure S7 shows Bayesian phylogenetic trees for a subset of the detected instances of horizontal gene transfer from the group with at least 1 gain and 1 loss (No. 1 to 10) and the non-orthologous groups (No. 11 to 15). The scale bar represents [file 1471-2148-12-243-S2.ZIP › Additional data file 2 revised/Figure S3.png]

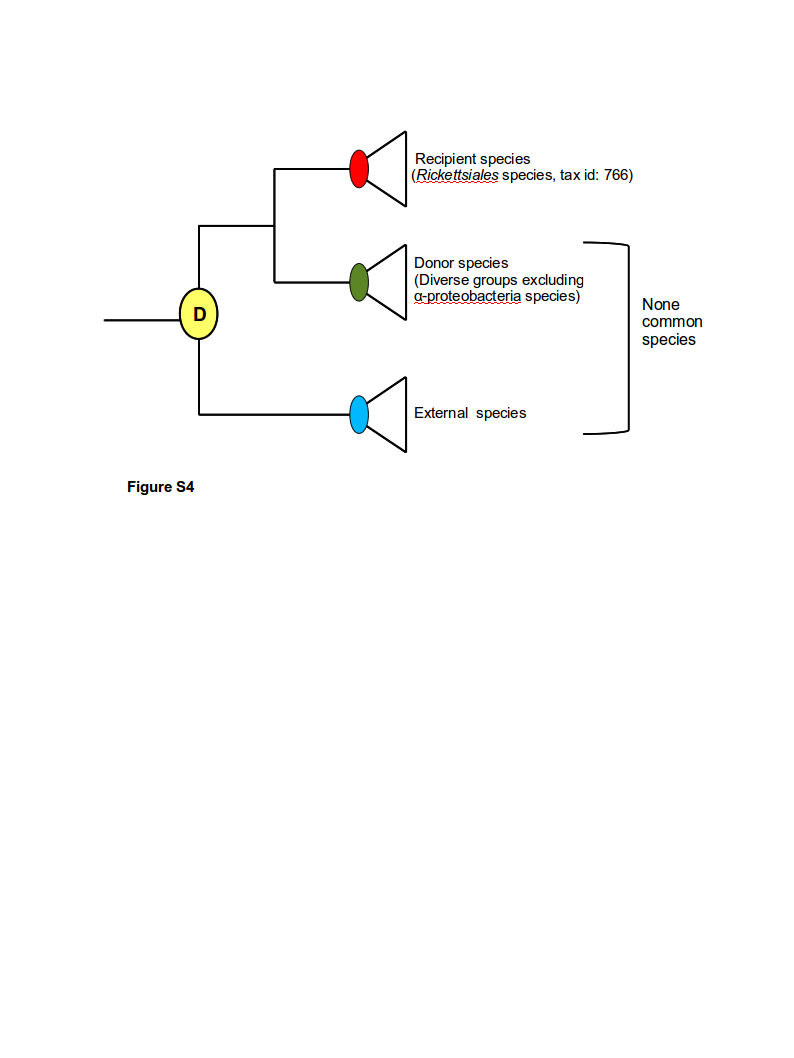

Supplement: Additional file 2 — Figure S1 shows the 16S rRNA species tree.Figure S2 shows the two principle patterns for the detection of gene gain and gene loss using PhyloPattern: a. Loss event and b. Gain event. Red circles represent the “present” characteristic, blue circles represent the “absent” characteristic. The pattern was defined as a loss event if the gene was present in the ancestral node and one descendant node but absent in another node (violet rhomboid). Conversely, the pattern was defined as a gain event if the gene was absent in the ancestral node and one descendant node but was present in another descendant node (green rhomboid). Figure S3 maps the gain and loss events from the phyletic patterns onto the species tree. Red circles represent the “present” characteristic, and blue circles represent the “absent” characteristic. Violet and green rhomboids represent loss and gain events, respectively. Figure S4 shows the transfer pattern detected by HGT agent from the multi-agent system DAGOBAH. HGT agent applied PhyloPattern to annotate the subtree (see Methods). Yellow circle with a D indicates that a duplication of the gene occurred at that node. Red, green and cyan circles represent recipient species, donor species and external species, respectively. Tax id: taxonomy identity. Figure S5 shows the results of the number of orthologous groups obtained from the gene gain and gene loss analysis by PhyloPattern in each respective event. Figure S6a is a flowchart representing the detailed description of the systematic analysis of horizontal gene transfer detection with at least one gain event. Figure S6b is a flowchart representing the detailed description of the systematic analysis of horizontal gene transfer detection for non-orthologous groups. Figure S7 shows Bayesian phylogenetic trees for a subset of the detected instances of horizontal gene transfer from the group with at least 1 gain and 1 loss (No. 1 to 10) and the non-orthologous groups (No. 11 to 15). The scale bar represents [file 1471-2148-12-243-S2.ZIP › Additional data file 2 revised/Figure S4.png]

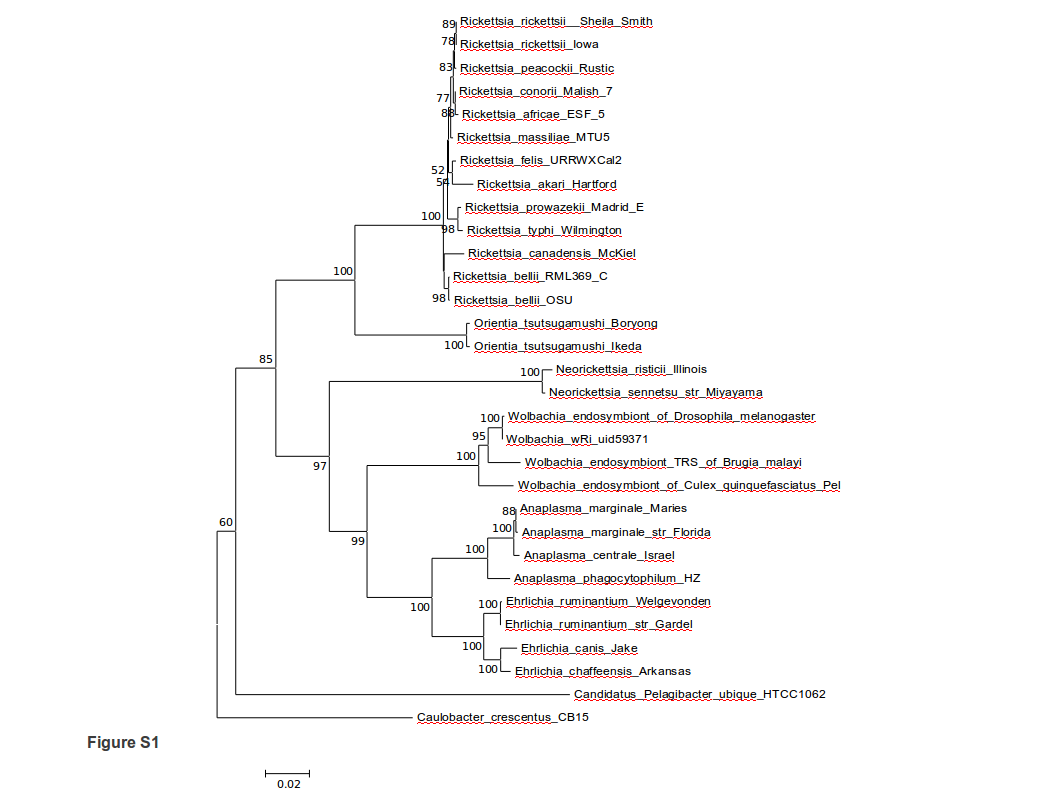

Supplement: Additional file 2 — Figure S1 shows the 16S rRNA species tree.Figure S2 shows the two principle patterns for the detection of gene gain and gene loss using PhyloPattern: a. Loss event and b. Gain event. Red circles represent the “present” characteristic, blue circles represent the “absent” characteristic. The pattern was defined as a loss event if the gene was present in the ancestral node and one descendant node but absent in another node (violet rhomboid). Conversely, the pattern was defined as a gain event if the gene was absent in the ancestral node and one descendant node but was present in another descendant node (green rhomboid). Figure S3 maps the gain and loss events from the phyletic patterns onto the species tree. Red circles represent the “present” characteristic, and blue circles represent the “absent” characteristic. Violet and green rhomboids represent loss and gain events, respectively. Figure S4 shows the transfer pattern detected by HGT agent from the multi-agent system DAGOBAH. HGT agent applied PhyloPattern to annotate the subtree (see Methods). Yellow circle with a D indicates that a duplication of the gene occurred at that node. Red, green and cyan circles represent recipient species, donor species and external species, respectively. Tax id: taxonomy identity. Figure S5 shows the results of the number of orthologous groups obtained from the gene gain and gene loss analysis by PhyloPattern in each respective event. Figure S6a is a flowchart representing the detailed description of the systematic analysis of horizontal gene transfer detection with at least one gain event. Figure S6b is a flowchart representing the detailed description of the systematic analysis of horizontal gene transfer detection for non-orthologous groups. Figure S7 shows Bayesian phylogenetic trees for a subset of the detected instances of horizontal gene transfer from the group with at least 1 gain and 1 loss (No. 1 to 10) and the non-orthologous groups (No. 11 to 15). The scale bar represents [file 1471-2148-12-243-S2.ZIP › Additional data file 2 revised/Figure S1.png]

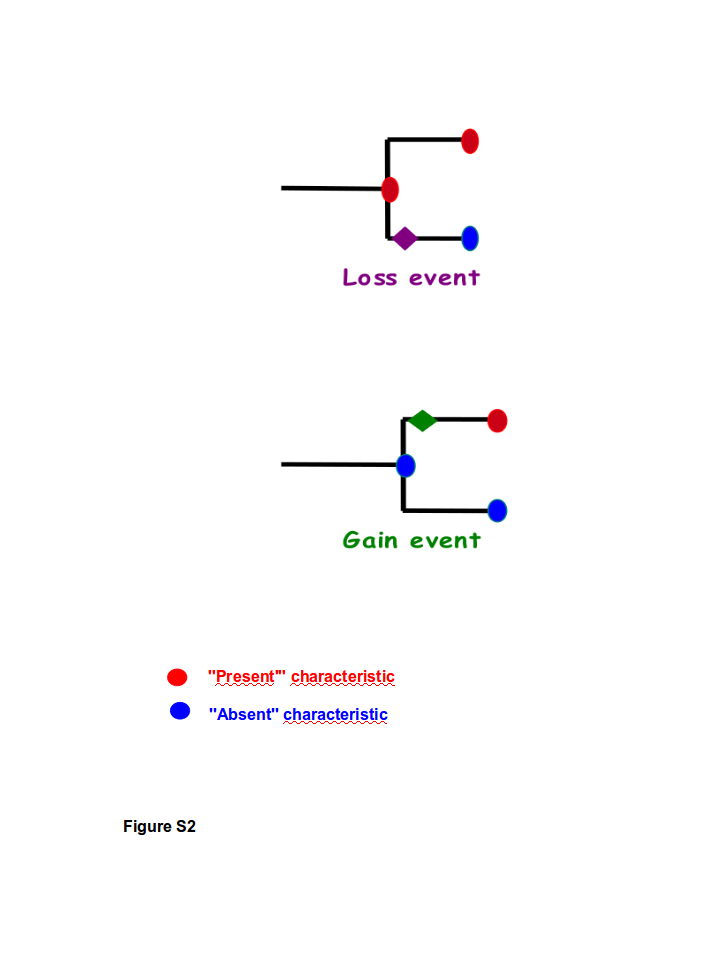

Supplement: Additional file 2 — Figure S1 shows the 16S rRNA species tree.Figure S2 shows the two principle patterns for the detection of gene gain and gene loss using PhyloPattern: a. Loss event and b. Gain event. Red circles represent the “present” characteristic, blue circles represent the “absent” characteristic. The pattern was defined as a loss event if the gene was present in the ancestral node and one descendant node but absent in another node (violet rhomboid). Conversely, the pattern was defined as a gain event if the gene was absent in the ancestral node and one descendant node but was present in another descendant node (green rhomboid). Figure S3 maps the gain and loss events from the phyletic patterns onto the species tree. Red circles represent the “present” characteristic, and blue circles represent the “absent” characteristic. Violet and green rhomboids represent loss and gain events, respectively. Figure S4 shows the transfer pattern detected by HGT agent from the multi-agent system DAGOBAH. HGT agent applied PhyloPattern to annotate the subtree (see Methods). Yellow circle with a D indicates that a duplication of the gene occurred at that node. Red, green and cyan circles represent recipient species, donor species and external species, respectively. Tax id: taxonomy identity. Figure S5 shows the results of the number of orthologous groups obtained from the gene gain and gene loss analysis by PhyloPattern in each respective event. Figure S6a is a flowchart representing the detailed description of the systematic analysis of horizontal gene transfer detection with at least one gain event. Figure S6b is a flowchart representing the detailed description of the systematic analysis of horizontal gene transfer detection for non-orthologous groups. Figure S7 shows Bayesian phylogenetic trees for a subset of the detected instances of horizontal gene transfer from the group with at least 1 gain and 1 loss (No. 1 to 10) and the non-orthologous groups (No. 11 to 15). The scale bar represents [file 1471-2148-12-243-S2.ZIP › Additional data file 2 revised/Figure S2.png]

Figure S7:

1.157827021

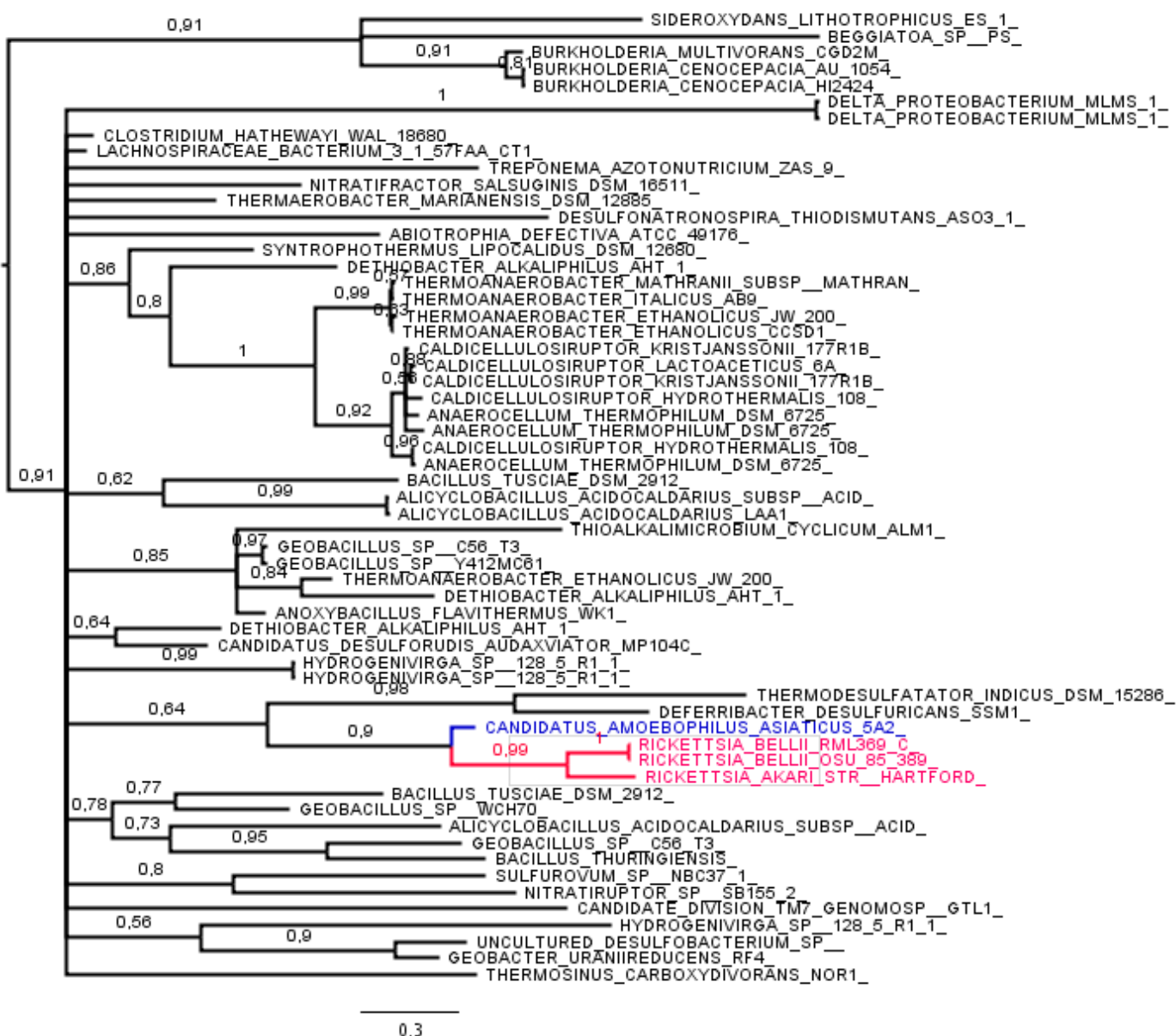

2.157826843

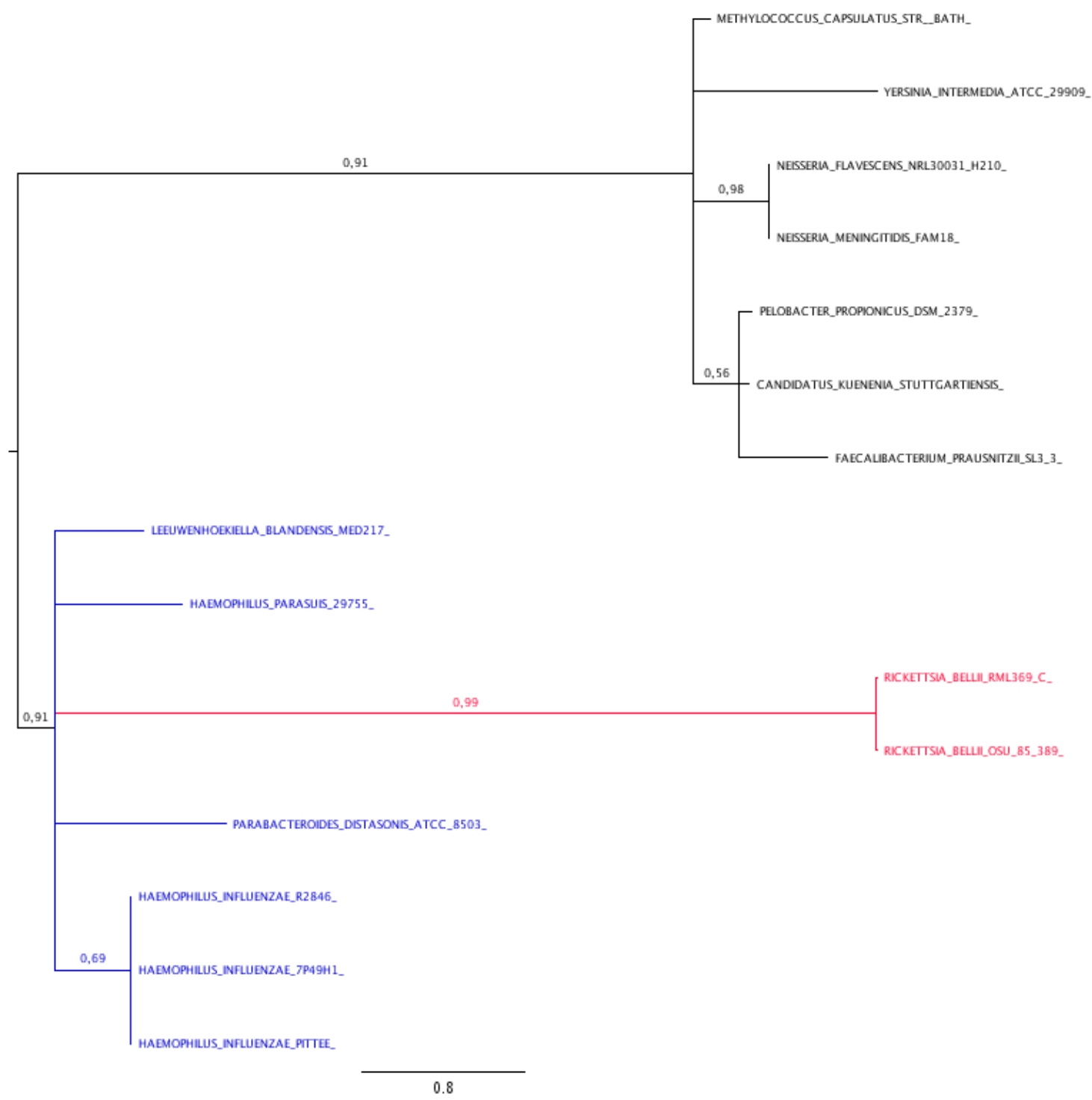

3. 225630595

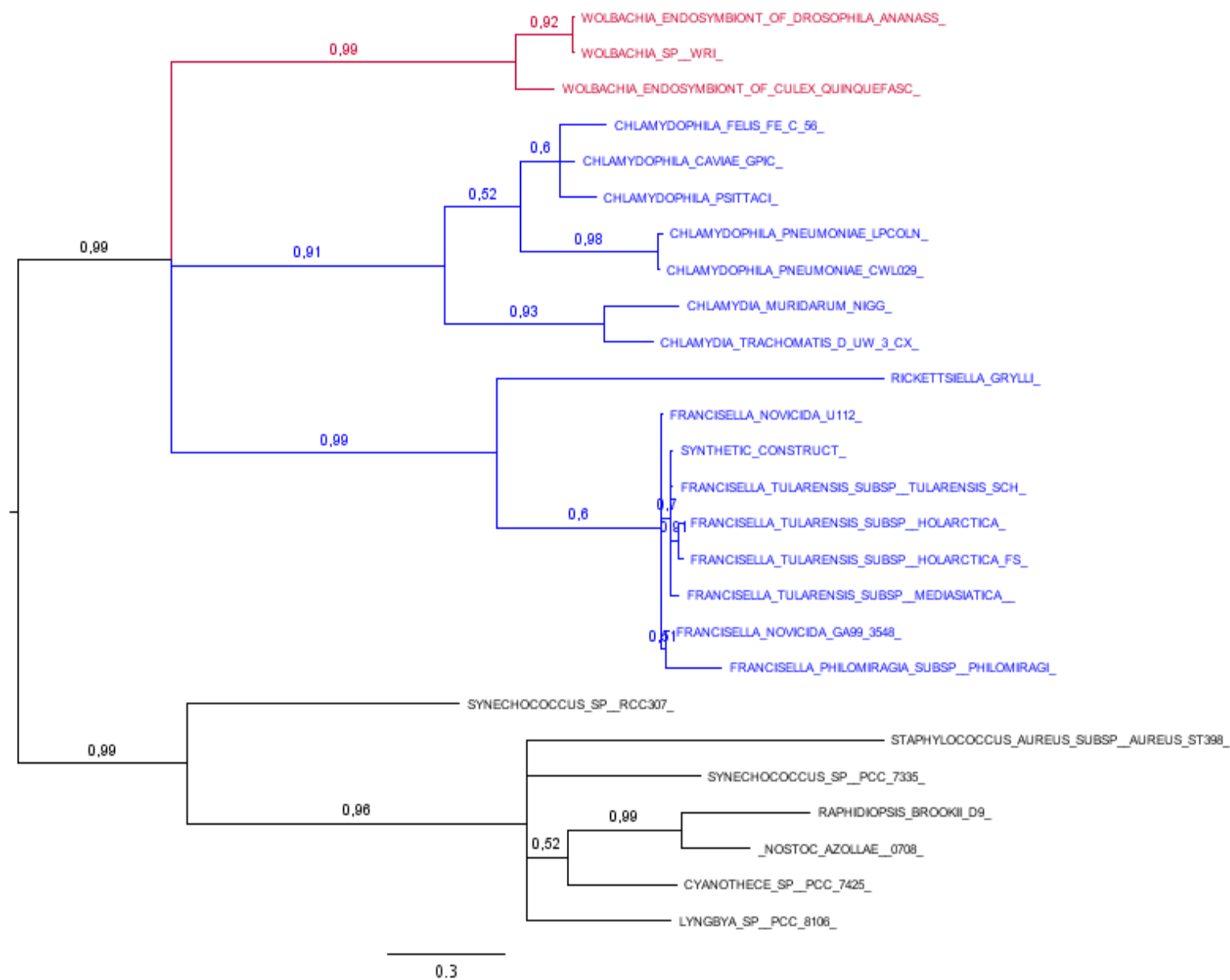

4. 157827797

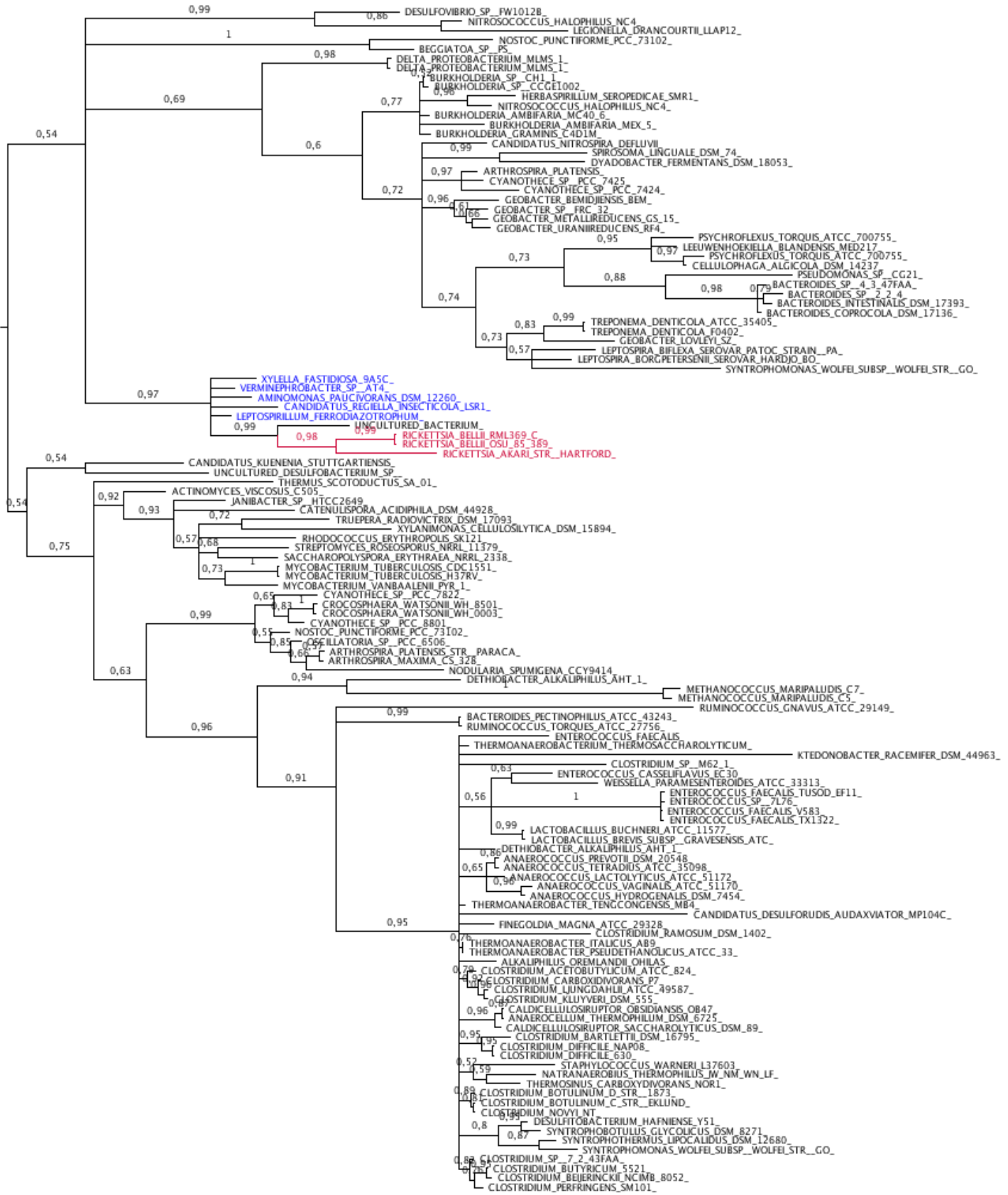

0.6

5. 225630592

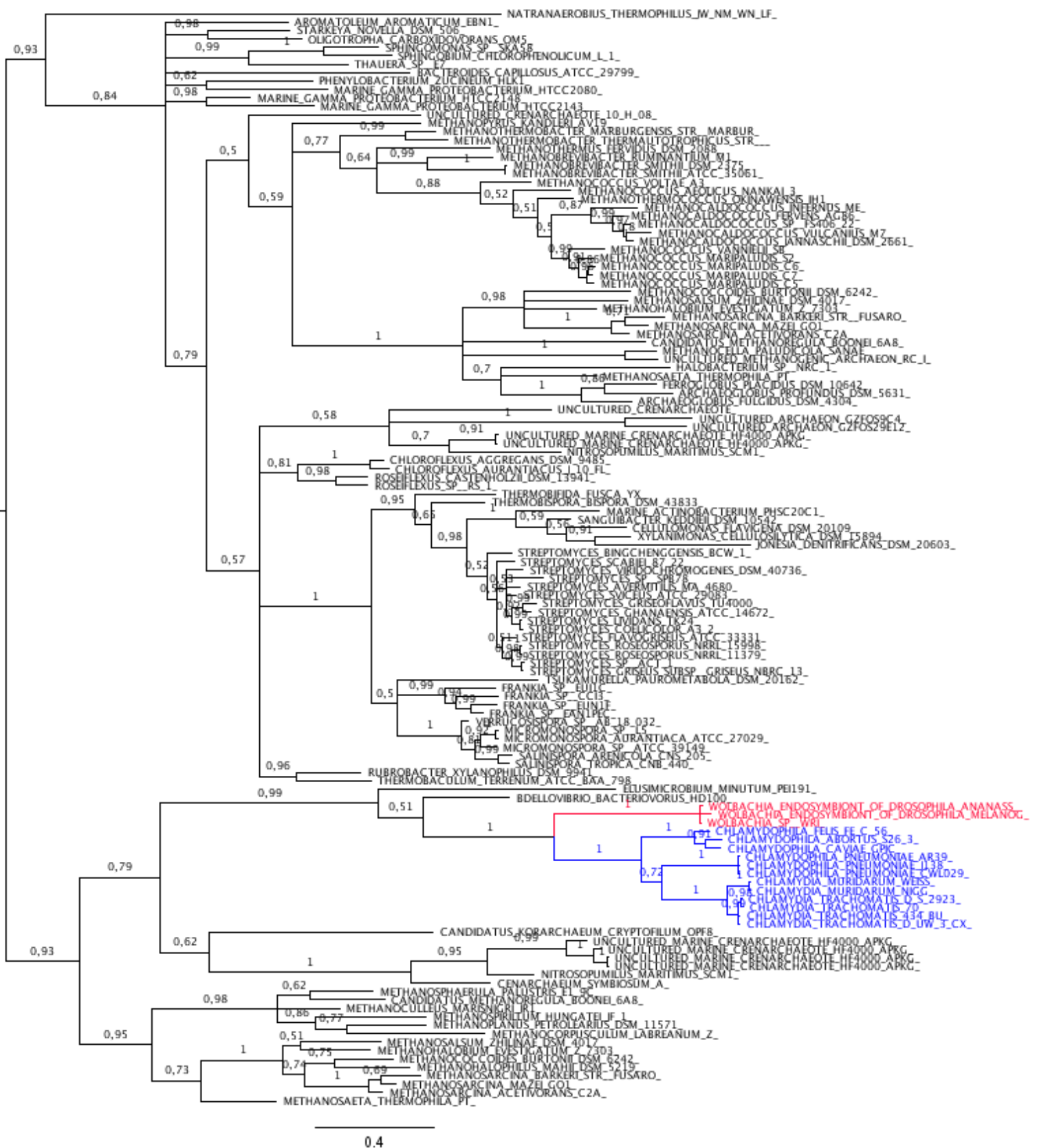

6. 42520378

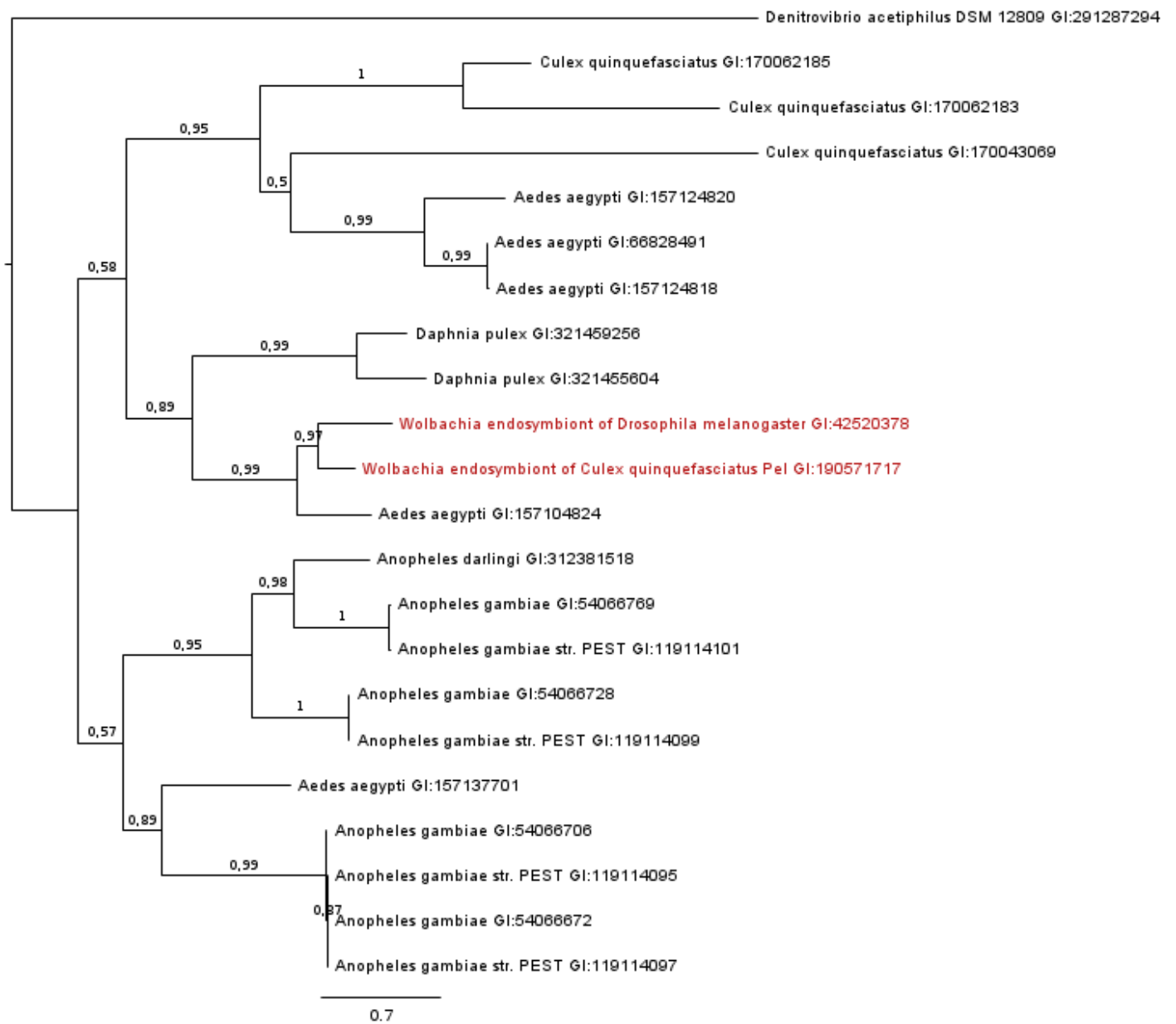

7. 238651161

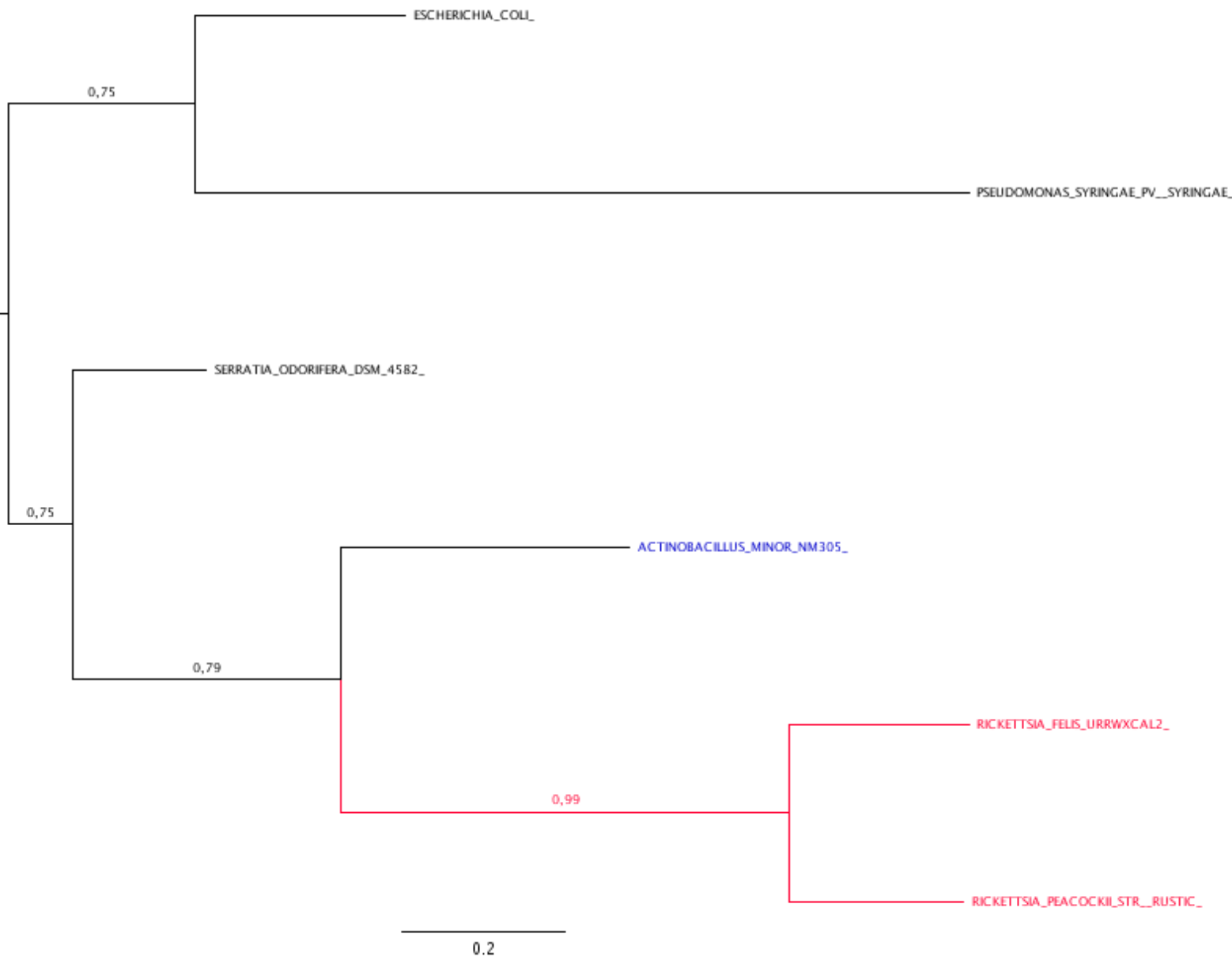

8. 189182975

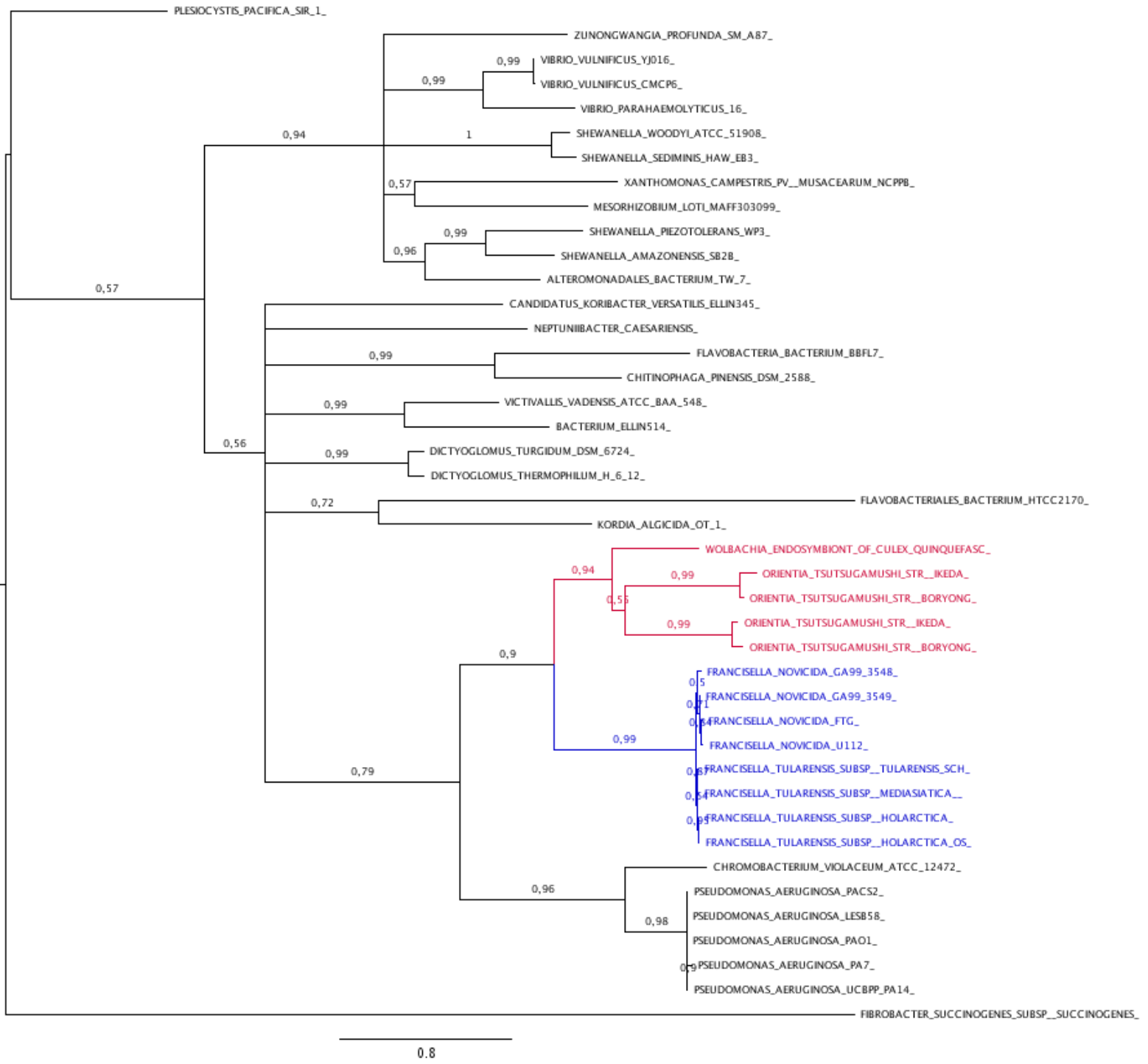

9. 157826324

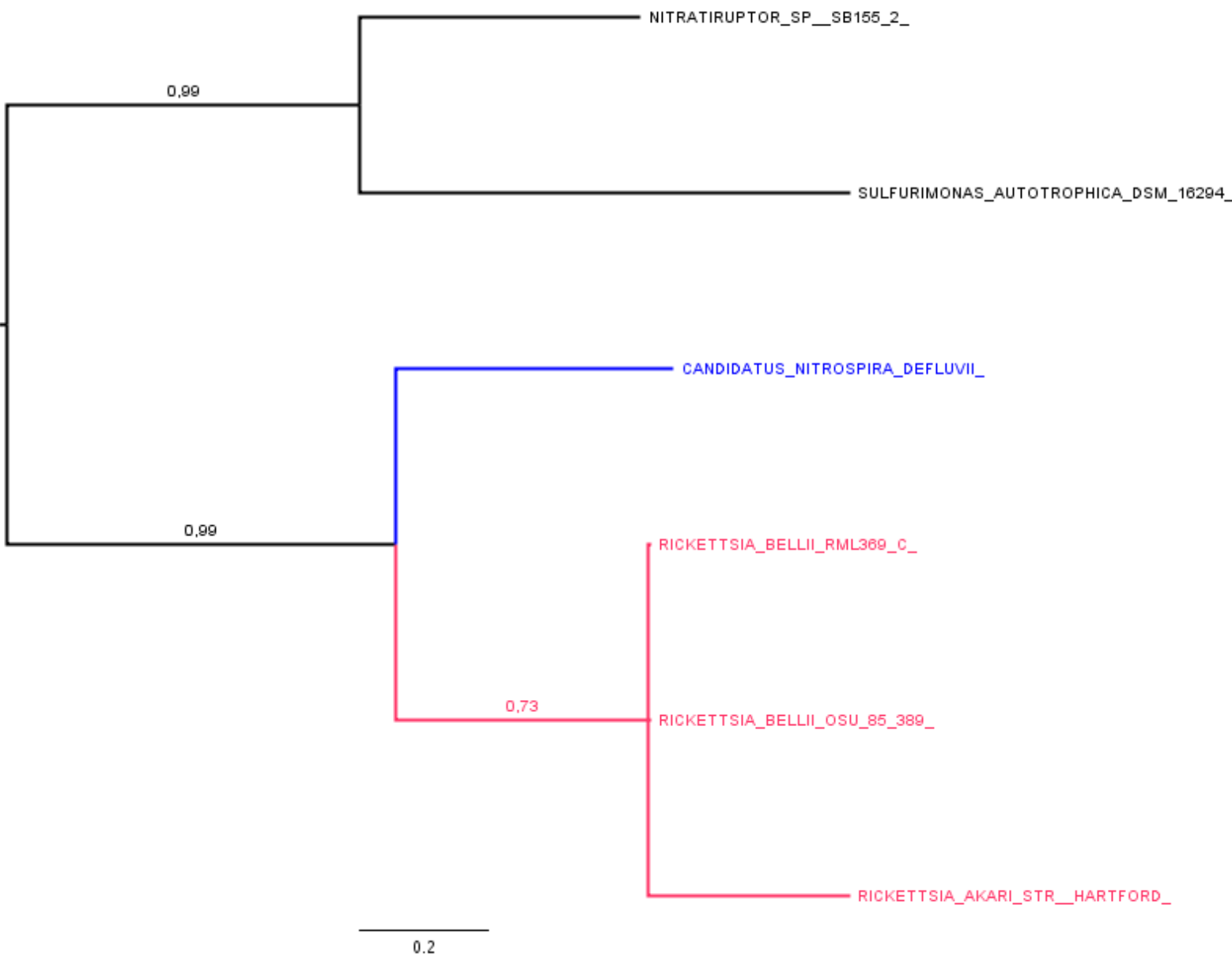

10. 157964120

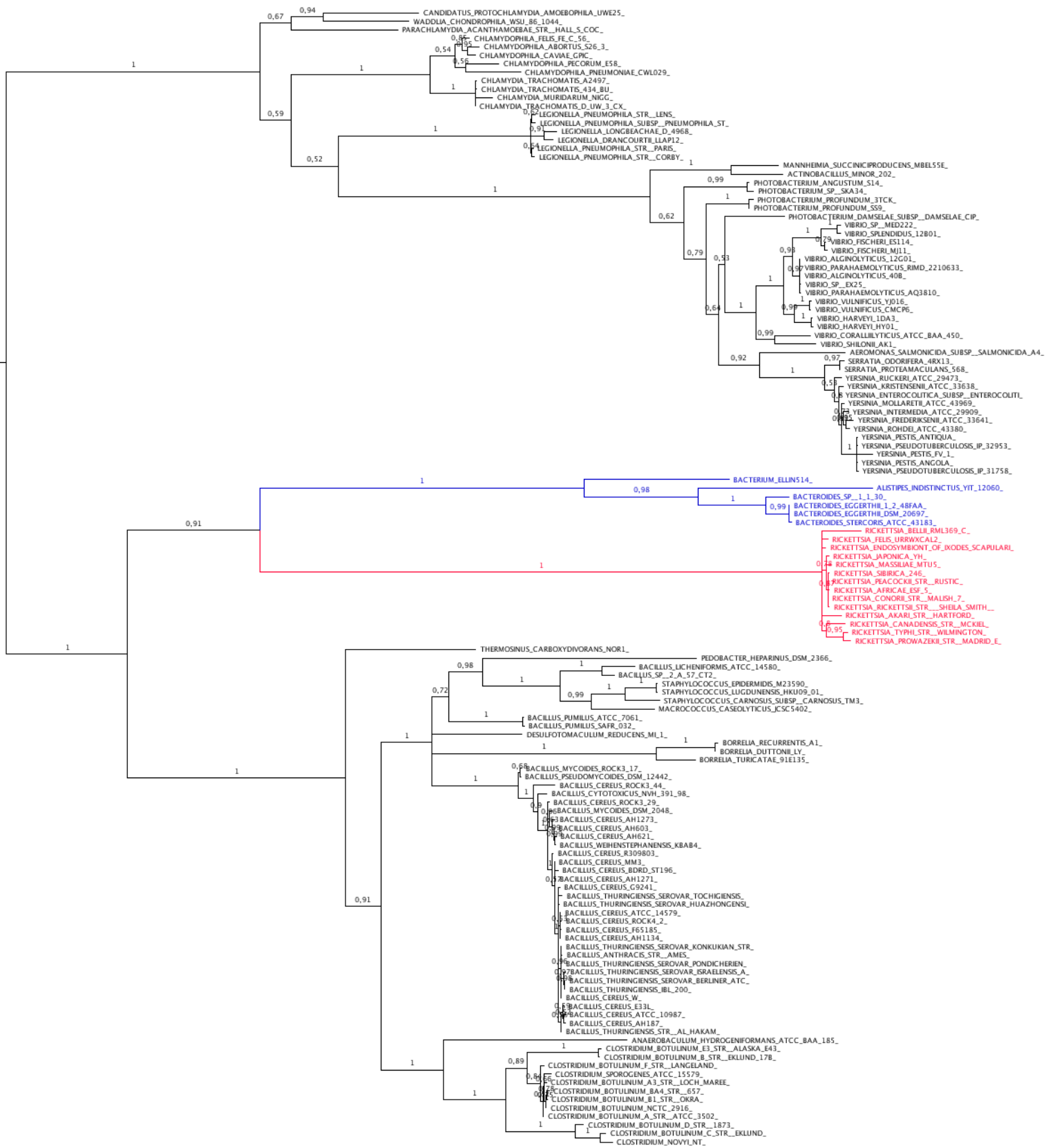

0.5

11. 91205673

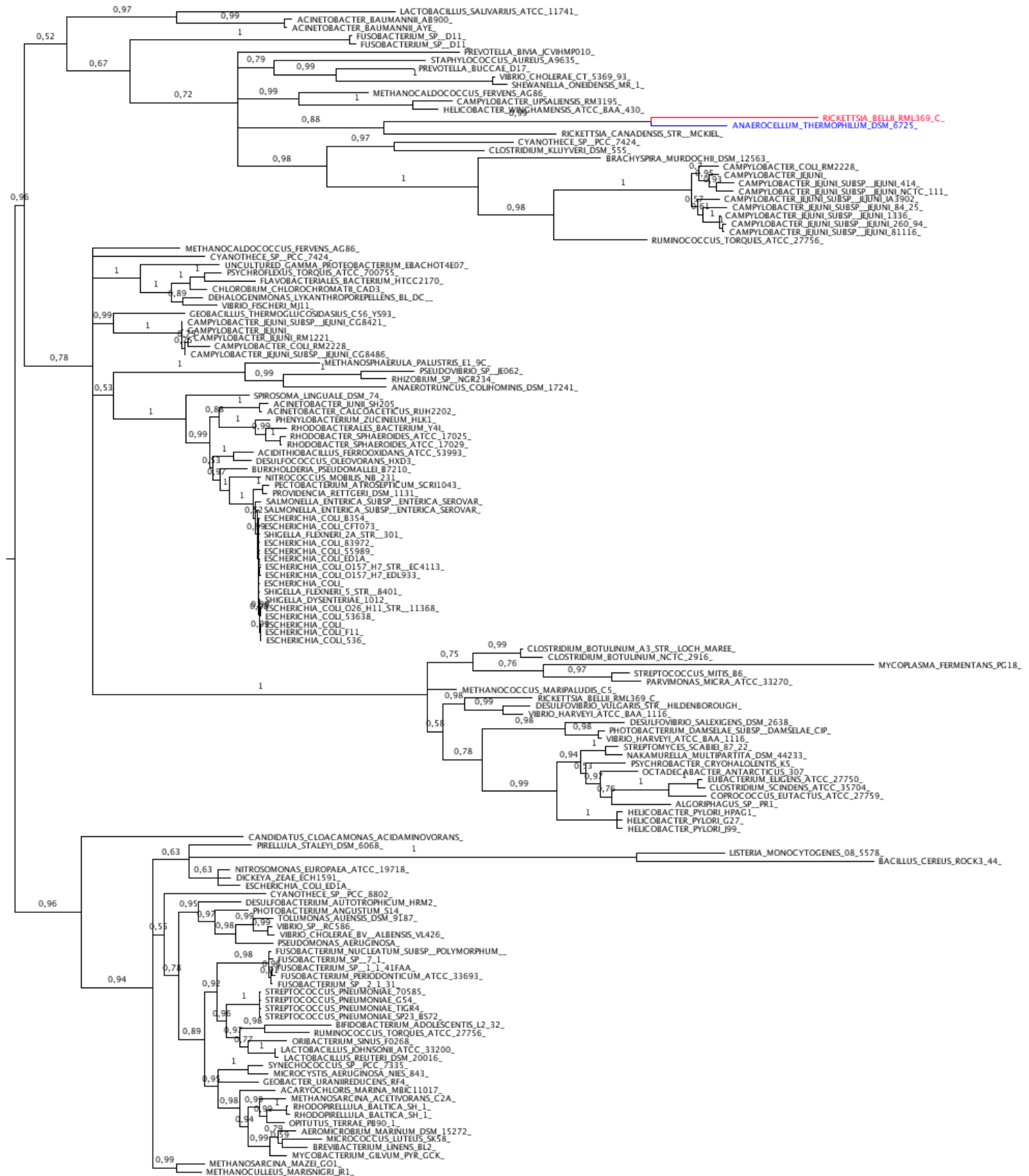

12. 71083820

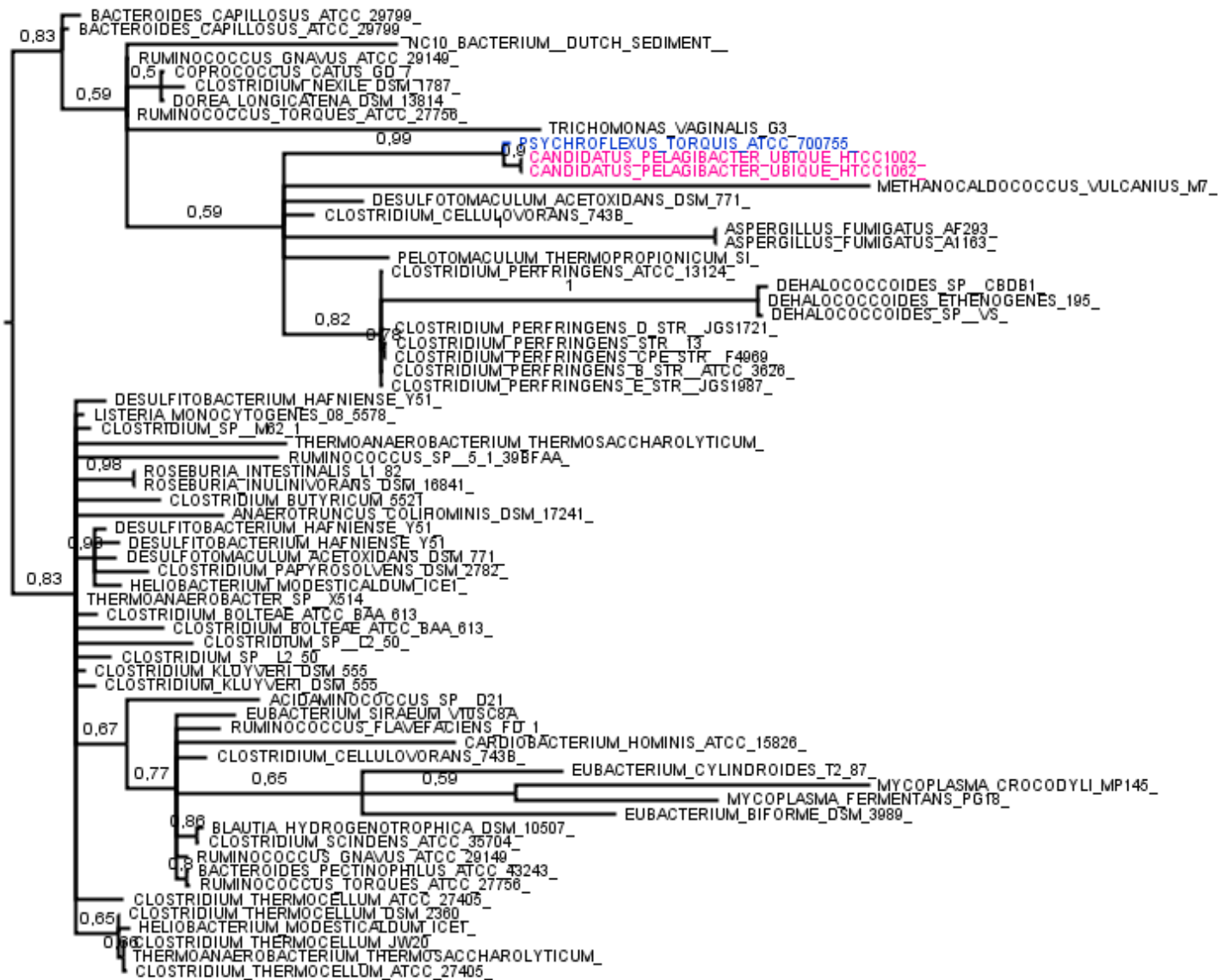

0.5

13. 71083892

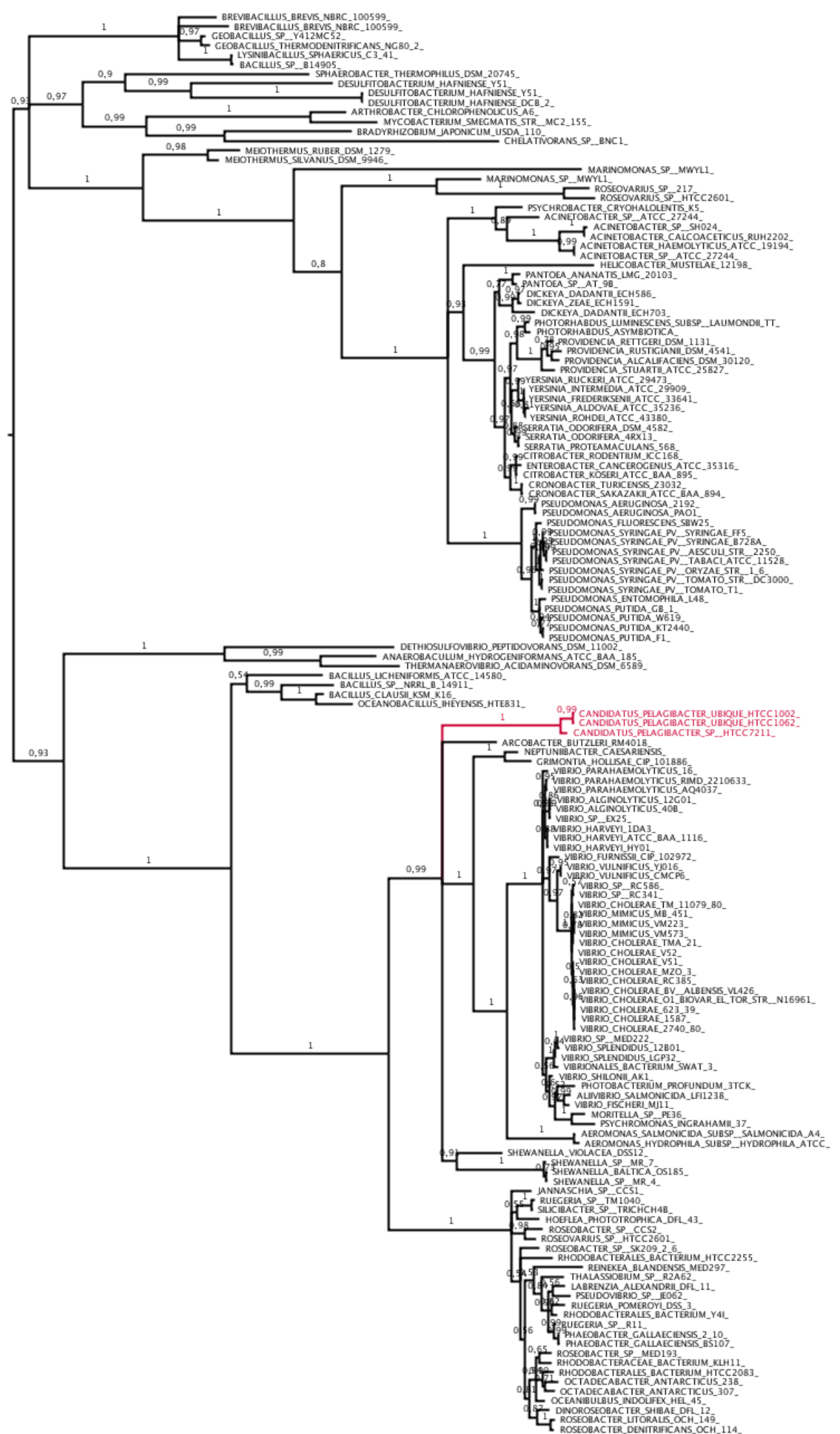

14. 71083916

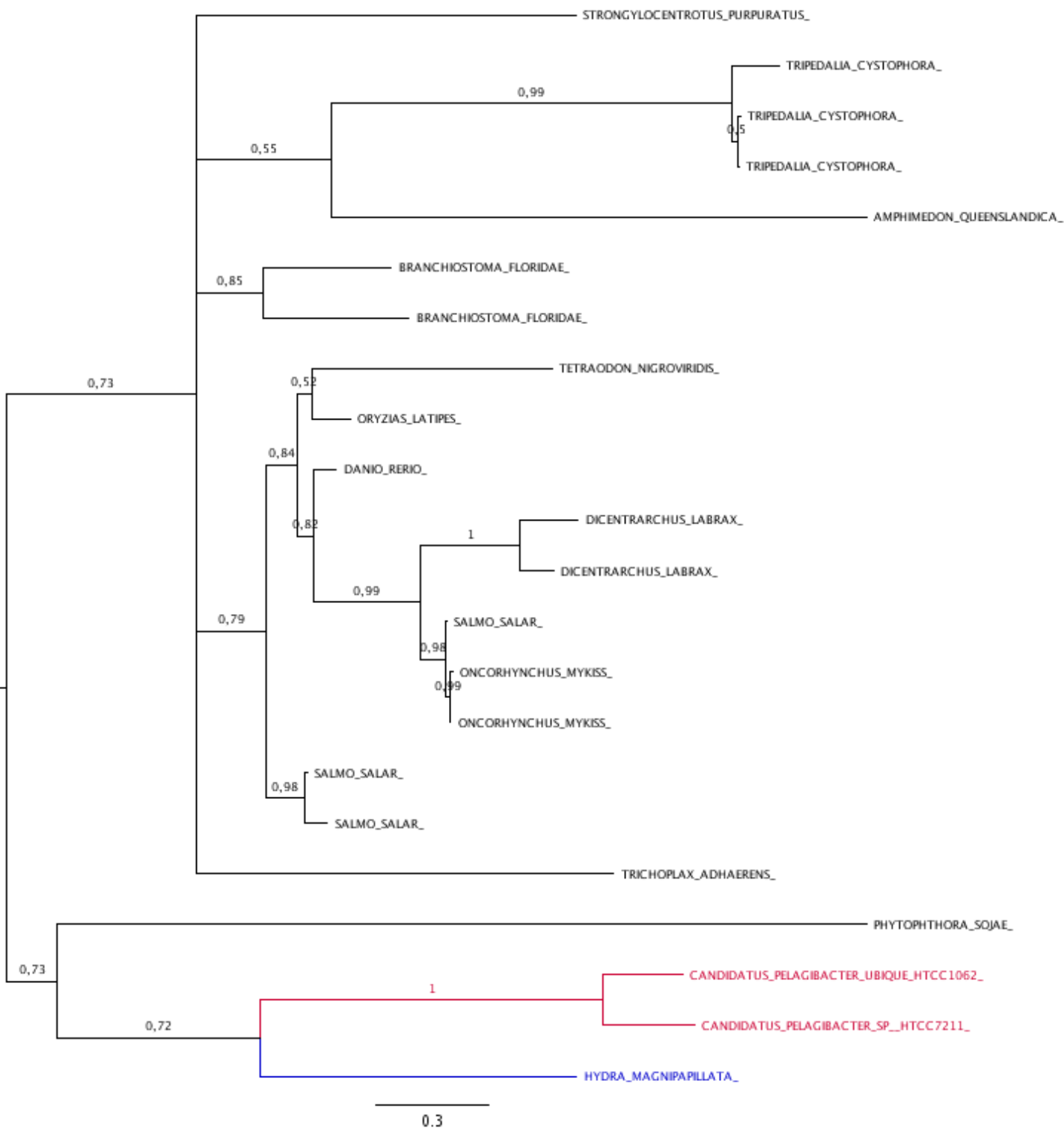

15. 42520763

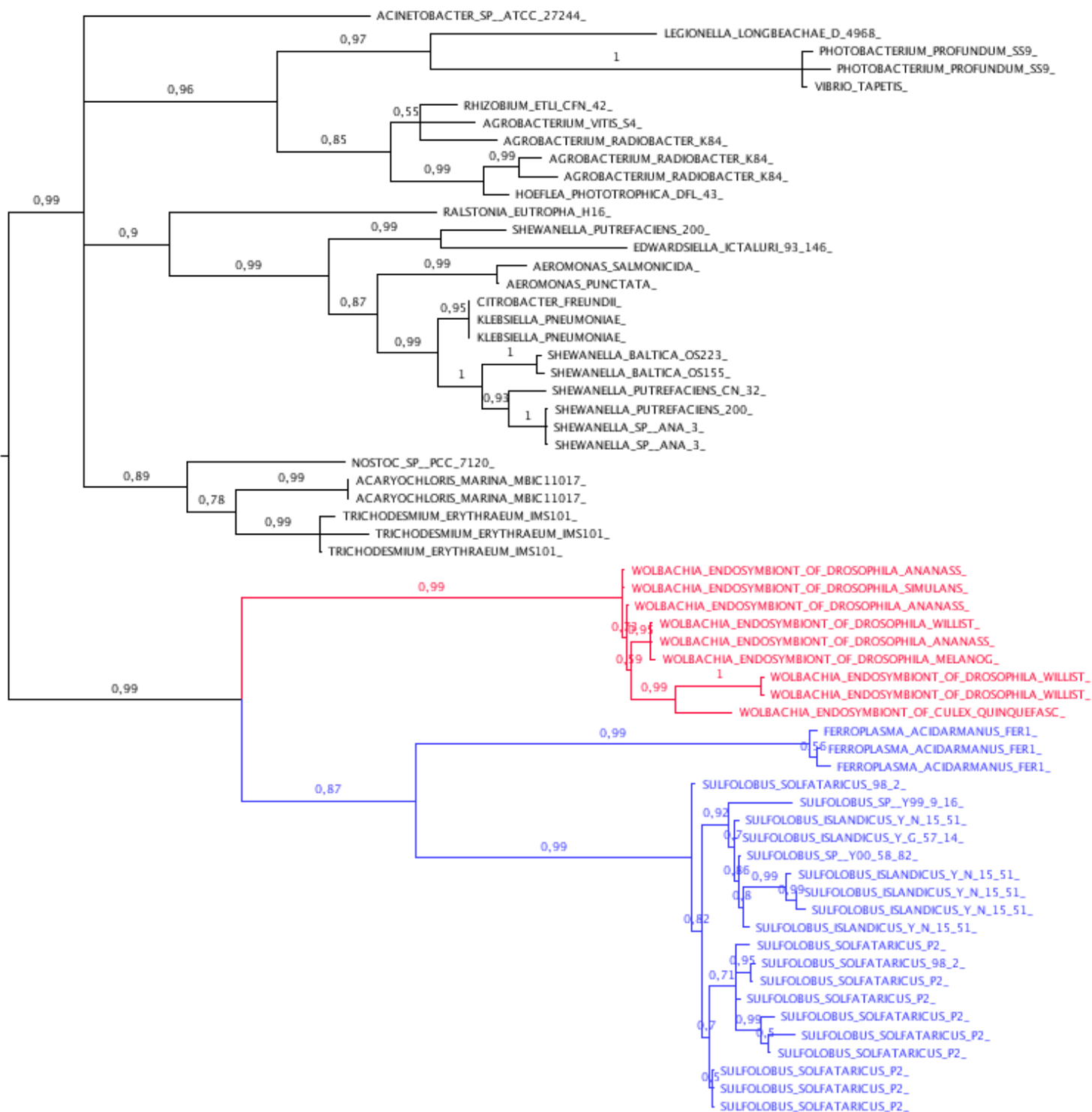

0.3

Supplement: Additional file 2 — Figure S1 shows the 16S rRNA species tree.Figure S2 shows the two principle patterns for the detection of gene gain and gene loss using PhyloPattern: a. Loss event and b. Gain event. Red circles represent the “present” characteristic, blue circles represent the “absent” characteristic. The pattern was defined as a loss event if the gene was present in the ancestral node and one descendant node but absent in another node (violet rhomboid). Conversely, the pattern was defined as a gain event if the gene was absent in the ancestral node and one descendant node but was present in another descendant node (green rhomboid). Figure S3 maps the gain and loss events from the phyletic patterns onto the species tree. Red circles represent the “present” characteristic, and blue circles represent the “absent” characteristic. Violet and green rhomboids represent loss and gain events, respectively. Figure S4 shows the transfer pattern detected by HGT agent from the multi-agent system DAGOBAH. HGT agent applied PhyloPattern to annotate the subtree (see Methods). Yellow circle with a D indicates that a duplication of the gene occurred at that node. Red, green and cyan circles represent recipient species, donor species and external species, respectively. Tax id: taxonomy identity. Figure S5 shows the results of the number of orthologous groups obtained from the gene gain and gene loss analysis by PhyloPattern in each respective event. Figure S6a is a flowchart representing the detailed description of the systematic analysis of horizontal gene transfer detection with at least one gain event. Figure S6b is a flowchart representing the detailed description of the systematic analysis of horizontal gene transfer detection for non-orthologous groups. Figure S7 shows Bayesian phylogenetic trees for a subset of the detected instances of horizontal gene transfer from the group with at least 1 gain and 1 loss (No. 1 to 10) and the non-orthologous groups (No. 11 to 15). The scale bar represents [file 1471-2148-12-243-S2.ZIP › Additional data file 2 revised/Figure S7.pdf]
