# Supplementary material for: An automated approach for the identification of horizontal gene transfers from complete genomes reveals the rhizome of Rickettsiales
Source: BMC Evol Biol. 2012 Dec 12;12:243. doi: 10.1186/1471-2148-12-243 (PMC3575314; doi:10.1186/1471-2148-12-243)
Supplement: Additional file 1 — Table S1 shows the NCBI accession numbers for the studied species.Table S2 shows the Bayesian analysis for the horizontal gene transfer cases from other species to Rickettsiales excluding alphaproteobacteria. Violet rows represent transfer cases from at least 1 gain event, and the brown rows represent transfer cases from a non-orthologous group. PP indicates the posterior probability value from the Bayesian analysis, aa indicates amino acids, and BV indicates the bootstrap values obtained with the NJ, MP, and ML methods. Table S3 is a list of annotations for the conserved genes from 267 Ortholog clusters of Rickettsiales and Caulobacter cresentus CB15 from the COG database. Table S4 is a list of annotations for the conserved genes from 277 Ortholog clusters of Rickettsia, Orientia, Wolbachia, Anaplasma, Ehrlichia, Neorickettsia and Candidatus Pelagibacter ubique from the COG database. Table S5 is a list of annotation for the conserved genes from 328 Ortholog clusters of Rickettsia, Orientia, Wolbachia, Anaplasma, Ehrlichia and Neorickettsia from the COG database. Table S6 is a list of annotation sfor the 1,709 orthologous groups based on the COG database. Table S7 is a table of the horizontal gene transfer cases identified from at least 1 gain and 1 loss event (part 1). Table S8 is a table of the horizontal gene transfer cases identified from at least 1 gain and 1 loss event (part 2). Table S9 is a table of the horizontal gene transfer cases identified from non-orthologous groups. Table S10 is a table showing the distribution of proteins annotated from the Gene Ontology database. Table S11 is a table showing the assignment of proteins annotated by Gene Ontology at the 9th level. Table S12 is a table showing the assignment of proteins annotated by Gene Ontology at the 12th level. Table S13 is a table showing the non-synonymous/synonymous substitution analysis for de novo genes. [file 1471-2148-12-243-S1.ZIP › Additional data file 1 revised/Table S2.doc]

**Table S2: Table showing the Bayesian analysis for the horizontal transfer cases from other species to *Rickettsiales* excluding α-proteobacteria**

|  | Identity of gene | Donor species | Recipient species | Protein annotation from NCBI | Length of protein (aa) | BT | PP |
| --- | --- | --- | --- | --- | --- | --- | --- |
| 1 | 157827021 | *Candidatus* Amoebophilus asiaticus 5a2  (Bacteroidetes) | *Rickettsia akari* str. Hartford  *Rickettsia bellii* OSU 85-389  *Rickettsia bellii* RML 369-C | Unnamed product protein | 130 | 99-90-50 | 0.9 |
| 2 | 157826843 | *Haemophilus parasuis* 29755  *Leeuwenhoekiella blandensis* MED217  *Haemophilus influenzae* pittee  *Haemophilus influenzae* R2846  *Haemophilus influenzae* 7p49h1  *Parabacteroides distasonis* ATCC 8503  (Gamma-proteobacteria) | *Rickettsia bellii* OSU 85-389  *Rickettsia bellii* RML 369-C | Unnamed product protein | 202 | 99-*-* | 0.91 |
| 2 | 225630595 | *Francisella tularensis*  (Gamma-proteobacteria) | *Wolbachia* ensosymbiont of Culex quinquefasciatus Pel  *Wolbachia* sp. wRi  *Wolbachia* endosymbiont of Drosophila ananassae | Dihydroneopterin aldolase | 128 | 99-*-* | 0.99 |
| 4 | 157827797 | *Aminomonas paucivorans* DSM 12260  (Synergistetes)  *Leptospirillum ferrodiazotrophum* (Nitrospirales)  *Verminephrobacter* sp. AT4  (Beta proteobacteria)  *Xylella fastidiosa* 9A5C  (Gamma proteobacteria) | *Rickettsia akari* str. Hartford  *Rickettsia bellii* OSU 85-389  *Rickettsia bellii* RML 369-C | PemK-like growth inhibitor | 108 | 93-60-64 | 0.99 |
| 5 | 225630592 | *Chlamydophila pneumoniae* CWL029  *Chlamydophila pneumoniae* AR39  *Chlamydophila pneumoniae* J138  (Chlamydiae) | *Wolbachia* endosymbiont of Drosophila melanogaster | Hypothetical protein | 251 | 91-53-72 | 1 |
| 6 | 225630748 | *Pseudomonas fluoresence* SB W25  *Plesiocystis pacifica* SI R1  *Haliangium ochraceum* DSM 143-65 | *Rickettsia* endosymbiont of Ixodes scapularis  *Wolbachia* sp. wRi  *Wolbachia* endosymbiont of Drosophila simulans | Hypothetical protein | 329 | 100-100-98 | 0.85 |
| 7 | 238651161 | *Actinobacillus minor* NM305  (Gamma proteobacteria) | *Rickettsia felis* URRWXCal2  *Rickettsia peacockii* str. Rustic | Hypothetical protein | 82 | 48-96-* | 0.79 |
| 8 | 189182975 | *Francisella novicida* GA99-3548  *Francisella tularensis* subsp. holarctica  *Francisella tularensis* subsp. holarctica OSU18  *Francisella tularensis*subsp. tularensis SCHU S4  *Francisella tularensis* subsp. mediasiatica FSC147  *Francisella novicida* GA99-3549  *Francisella novicida* U112  *Francisella novicida* FTG  (Gamma proteobacteria) | *Orientia tsutsugamushi* str. Boryong  *Orientia tsutsugamushi* str. Ikeda  *Wolbachia* endosymbiont of Culex quinquefasciatus Pel | Hypothetical protein | 632 | 100-95-* | 0.9 |
| 9 | 157826324 | *Candidatus* Nitrospira defluvii  (Nitrospirales) | *Rickettsia akari* str. Hartford  *Rickettsia bellii* OSU 85-389  *Rickettsia bellii* RML 369-C | Growth inhibitor | 88 | 99-*-100 | 0.99 |
| 10 | 157964120 | *Bacterium* ellin514  *Bacteroides* sp. 1_1_30  *Bacteroides eggerthii* 1_2_48faa  *Bacteroides eggerthii* DSM 20697  *Bacteroides stercoris* ATCC 43183  *Alistipes indistinctus* YIT 12060 (Firmicutes) | *Rickettsia bellii* RML 369-C  *Rickettsia canadensis* str Mckiel  *Rickettsia* endosymbiont of Ixodes scapularis  *Rickettsia felis* URRWXCal2  *Rickettsia japonica* YH  *Rickettsia sibirica* 246  *Rickettsia africae* ESF-5  *Rickettsia peacockii* str. Rustic  *Rickettsia conorii* str. Malish 7  *Rickettsia rickettsii* str. 'Sheila Smith'  *Rickettsia massiliae* Mtu5  *Rickettsia akari* str. Hartford  *Rickettsia prowazekii* str. Madrid E  *Rickettsia typhi* str. Wilmington | Sugar phosphate permease | 439 | 92-100-* | 0.91 |
| 11 | 91205673 | *Anaerocellum thermophilum* DSM 6725  (Firmicutes) | *Rickettsia bellii* RML369-C | unnamed protein product | 540 | 100-100-99 | 0.88 |
| 12 | 71083820 | *Psychroflexus* *torquis* ATCC 700755 (Bacteroidetes) | *Candidatus* Pelagibacter ubique HTCC1062 | unnamed protein product | 133 | 100-100-74 | 0.99 |
| 13 | 71083892 | *Shewanella violacea* DSS12  *Arcobacter butzleri* RM4018 (epsilon)  *Grimontia hollisae* CIP101886  *Neptuniibacter caesariensis*  *Psychromonas ingrahamii* 37  *Moritella* sp. PE36  *Photobacterium profundum* 3TCK  *Vibrio* spp.  *Aeromonas salmonicida* subsp. salmonicida A449  *Aeromonas hydrophila* subsp. hydrophila ATCC 7966  *Shewanella* sp. MR-7  *Shewanella baltica* OS185  *Shewanella* sp. MR-4  (Gamma proteobacteria) | *Candidatus* Pelagibacter sp HTCC7211  *Candidatus* Pelagibacter ubique HTCC1062 | tcta gene product | 500 | 75-67-70 | 0.99 |
| 14 | 71083916 | *Hydra magnipapillata*  (Metazoa) | *Candidatus* Pelagibacter sp. HTCC7211  *Candidatus* Pelagibacter ubique HTCC1062 | unnamed protein product | 317 | 86-100-* | 0.72 |
| 15 | 42520763 | *Sulfolobus* sp. Y99.9.16  *Sulfolobus* *islandicus* Y.G.57.14  *Sulfolobus* sp. Y00.58.82  *Sulfolobus* *islandicus* Y.N.15.51  *Sulfolobus* *solfataricus* P2  *Sulfolobus* *solfataricus* 98.2  *Ferroplasma* *acidarmanus* FER1 (Archaea) | Wolbachia endosymbiont of Drosophila simulans  Wolbachia endosymbiont of Drosophila ananassae  Wolbachia endosymbiont of Drosophila melanogaster  Wolbachia endosymbiont of Drosophila willistoni tsc#14030-0811 24  Wolbachia endosymbiont of Culex quinquefasciatus JHB | IS5 family transposase | 135 | 92-*-* | 0.99 |
