# Supplementary material for: An automated approach for the identification of horizontal gene transfers from complete genomes reveals the rhizome of Rickettsiales
Source: BMC Evol Biol. 2012 Dec 12;12:243. doi: 10.1186/1471-2148-12-243 (PMC3575314; doi:10.1186/1471-2148-12-243)
Supplement: Additional file 1 — Table S1 shows the NCBI accession numbers for the studied species.Table S2 shows the Bayesian analysis for the horizontal gene transfer cases from other species to Rickettsiales excluding alphaproteobacteria. Violet rows represent transfer cases from at least 1 gain event, and the brown rows represent transfer cases from a non-orthologous group. PP indicates the posterior probability value from the Bayesian analysis, aa indicates amino acids, and BV indicates the bootstrap values obtained with the NJ, MP, and ML methods. Table S3 is a list of annotations for the conserved genes from 267 Ortholog clusters of Rickettsiales and Caulobacter cresentus CB15 from the COG database. Table S4 is a list of annotations for the conserved genes from 277 Ortholog clusters of Rickettsia, Orientia, Wolbachia, Anaplasma, Ehrlichia, Neorickettsia and Candidatus Pelagibacter ubique from the COG database. Table S5 is a list of annotation for the conserved genes from 328 Ortholog clusters of Rickettsia, Orientia, Wolbachia, Anaplasma, Ehrlichia and Neorickettsia from the COG database. Table S6 is a list of annotation sfor the 1,709 orthologous groups based on the COG database. Table S7 is a table of the horizontal gene transfer cases identified from at least 1 gain and 1 loss event (part 1). Table S8 is a table of the horizontal gene transfer cases identified from at least 1 gain and 1 loss event (part 2). Table S9 is a table of the horizontal gene transfer cases identified from non-orthologous groups. Table S10 is a table showing the distribution of proteins annotated from the Gene Ontology database. Table S11 is a table showing the assignment of proteins annotated by Gene Ontology at the 9th level. Table S12 is a table showing the assignment of proteins annotated by Gene Ontology at the 12th level. Table S13 is a table showing the non-synonymous/synonymous substitution analysis for de novo genes. [file 1471-2148-12-243-S1.ZIP › Additional data file 1 revised/Table S13.doc]

**Table S13. Table showing the non-synonymous/synonymous substitution analysis for *De novo*** genes.

|  | **Gene identity number** | **Found in** | **Length**  **(nt)** | **Ka/Ks** | **P-value** | **Coding- region** | **GC%** |
| --- | --- | --- | --- | --- | --- | --- | --- |
| 1 | 56416688 | *Anaplasma marginale* str. St. Maries  *Anaplasma marginale* str. Florida  *Anaplasma marginale* str. Mississippi  *Anaplasma centrale* str. Israel | 1890  1893  381  1425 | 1.23  1.24  0.92  1.13 | 4.78e-10  9.01458e-11  0.27  0 | No | 51.4  52.4  43.6  51.7 |
| 2 | 73667082 | *Ehrlichia canis* str. Jake  *Ehrlichia chaffeensis* str. Sapulpa  *Ehrlichia chaffeensis* str. Arkansas | 1992  2199  2199 | 0.82  0.84  0.83 | 3.26e-07  1.01e-05  7.82e-07 | Yes | 27.0  28.3  28.4 |
| 3 | 58579406 | *Ehrlichia ruminantium* str. Welgevonden  *Ehrlichia ruminantium* str. Gardel  *Ehrlichia ruminantium*  *Ehrlichia canis* str. Jake | 2058  1758  573  2877 | 0.73  0.72  0.74  0.71 | 1.46e-15  1.27e-15  0  4.31e-32 | Yes | 32.5  32.0  32.3  33.4 |
| 4 | 56416615 | *Anaplasma marginale* str. St. Maries  *Anaplasma marginale* str. Florida  *Anaplasma marginale* str. Virginia  *Anaplasma centrale* str. Israel  *Anaplasma marginale* str. Mississippi | 2058  2061  2091  4422  216 | 1.01  1.06  1.11  1.16  1.21 | 0.81  0.05  0  1.30e-11  0.04 | No | 51.1  51.4  51.5  50.6  55.5 |
| 5 | 56416613 | *Anaplasma marginale* str. St. Maries  *Anaplasma marginale* str. Florida  *Anaplasma marginale* str. Puerto Rico  *Anaplasma centrale* str. Israel | 2304  2304  2304  2307 | 1.09  1.06  1.1  1.12 | 0  0  0.06  0 | No | 51.9  51.7  51.8  52.2 |
| 6 | 56416790 | *Anaplasma marginale* str. St. Maries  *Anaplasma marginale* str. Florida  *Anaplasma marginale* str. Puerto Rico  *Anaplasma marginale* str. Mississippi  *Anaplasma centrale* str. Israel | 2619  2556  1617  1371  2151 | 1.01  1.2  1.1  0.99  1.03 | 0.62  4.69382e-12  0.01  0.83  0.24 | No | 50.5  50.2  49.2  48.3  48.6 |
| 7 | 56416617 | *Anaplasma marginale* str. St. Maries  *Anaplasma marginale* str. Florida  *Anaplasma marginale* str. Puerto Rico  *Anaplasma marginale* str. Virginia  *Anaplasma centrale* str. Israel | 1518  1416  1470  1470  4422 | 1.07  1.18  1.09  1.08  1.06 | 0.05  2.38789e-05  0.02  0.04  1.30015e-11 | No | 49.6  49.3  49.5  49.6  50.6 |
| 8 | 56416857 | *Anaplasma phagocytophilum* HZ  *Anaplasma marginale* str. Florida  *Anaplasma marginale* str. Virginia  *Anaplasma centrale* str. Israel  *Anaplasma marginale* str. Mississippi | 5238  5238  5217  5325  117 | 1.0  1.1  1.12  1.14  0.92 | 0.01  6.72044e-07  1.38727e-08  3.29213e-12  0.48 | No | 52.3  52.2  52.3  53.2  51.1 |
| 9 | 58578724 | *Ehrlichia ruminantium* str. Welgevonden  *Ehrlichia ruminantium* str. Gardel  *Ehrlichia canis* str. Jake | 5337  5172  5571 | 0.69  0.67  0.72 | 4.52e-71  9.21e-94  2.55e-55 | Yes | 32.3  32.1  31.1 |
| 10 | 73667012 | *Ehrlichia canis* str. Jake  *Ehrlichia chaffeensis* str. Sapulpa  *Ehrlichia chaffeensis* str. Arkansas  *Ehrlichia ruminantium* str. Welgevonden  *Ehrlichia ruminantium* str. Gardel  *Anaplasma marginale* str. St. Maries  *Anaplasma marginale* str. Florida  *Anaplasma centrale* str. Israel  *Anaplasma phagocytophilum* HZ | 6366  3831  5289  4503  4488  4791  4791  4386  5058 | 0.72  1.25  0.92  0.64  0.68  1.04  1.04  0.92  0.87 | 7.75e-61  6.87e-18  8.00e-05  4.07e-102  1.42e-68  0.04  0.08  7.35e-05  2.34e-12 | No | 32.8  40.0  34.6  32.0  32.2  50.0  49.9  49.8  40.9 |

**Ka/Ks estimation:** We found several cases of lineage specific proteins in the studied group from Blast analysis. We presumed them to be as new genes that may have been created to perform novel function in the respective lineage specific genomes. We have retrieved the corresponding gene sequences of these protein sequences and estimated the pariwise ω=Ka/Ks values using KaKs_Calculator v1.2 using MA model [74]. KaKs_Calculator is a software package that calculates non-synonymous (Ka) and synonymous (Ks) substitution rates through model selection and model averaging. Sequences with ω < 1 likely represent a protein-coding exon. A Fischer test was used to determine whether ω ratios were significantly less than one. Therefore, when the P value was <0.05, the sequences may be under purifying selection.

The origin of new genes can result from many genetic events such as gene duplication, gene fission and fusion, lateral gene transfer, transposable elements (TEs) and *de novo* gene formation (Chandrasekaran and Betrán 2008). We found several cases of lineage specific proteins from different groups such as 1,413 proteins are retrieved from at least 1 gain and 1 loss event and 774 proteins are retrieved from non-orthologous group. These proteins are present exclusively in *Rickettsiales* and are lineage specific. Therefore, we have considered them to be as new genes (Figures S6a, S6b ). We have only analysed the 10 longest sequences from the total number of novel genes that have been reported here because the probability that a sequence will be non-coding increases as sequence length decreases. The corresponding gene sequences of 10 longest proteins from *Anaplasma* and *Ehrlichia* species are retrieved and analysed for their function. Based on the Ka/Ks ratio (ω = Ka/Ks = non-synomynous/synomynous substitutions) of 10 analysed sequences, 3 may be under purifying selection, given that their ω ratio is significantly less than 1 (p-value < 0.05). The ω ratio of other 7 cases was around 1 and they may not be under purifying selection. Further studies are needed to investigate the function of these novel genes.
