# Supplementary material for: An automated approach for the identification of horizontal gene transfers from complete genomes reveals the rhizome of Rickettsiales
Source: BMC Evol Biol. 2012 Dec 12;12:243. doi: 10.1186/1471-2148-12-243 (PMC3575314; doi:10.1186/1471-2148-12-243)
Supplement: Additional file 1 — Table S1 shows the NCBI accession numbers for the studied species.Table S2 shows the Bayesian analysis for the horizontal gene transfer cases from other species to Rickettsiales excluding alphaproteobacteria. Violet rows represent transfer cases from at least 1 gain event, and the brown rows represent transfer cases from a non-orthologous group. PP indicates the posterior probability value from the Bayesian analysis, aa indicates amino acids, and BV indicates the bootstrap values obtained with the NJ, MP, and ML methods. Table S3 is a list of annotations for the conserved genes from 267 Ortholog clusters of Rickettsiales and Caulobacter cresentus CB15 from the COG database. Table S4 is a list of annotations for the conserved genes from 277 Ortholog clusters of Rickettsia, Orientia, Wolbachia, Anaplasma, Ehrlichia, Neorickettsia and Candidatus Pelagibacter ubique from the COG database. Table S5 is a list of annotation for the conserved genes from 328 Ortholog clusters of Rickettsia, Orientia, Wolbachia, Anaplasma, Ehrlichia and Neorickettsia from the COG database. Table S6 is a list of annotation sfor the 1,709 orthologous groups based on the COG database. Table S7 is a table of the horizontal gene transfer cases identified from at least 1 gain and 1 loss event (part 1). Table S8 is a table of the horizontal gene transfer cases identified from at least 1 gain and 1 loss event (part 2). Table S9 is a table of the horizontal gene transfer cases identified from non-orthologous groups. Table S10 is a table showing the distribution of proteins annotated from the Gene Ontology database. Table S11 is a table showing the assignment of proteins annotated by Gene Ontology at the 9th level. Table S12 is a table showing the assignment of proteins annotated by Gene Ontology at the 12th level. Table S13 is a table showing the non-synonymous/synonymous substitution analysis for de novo genes. [file 1471-2148-12-243-S1.ZIP › Additional data file 1 revised/Table S11.doc]

| Assignment of GO terms at the 9^th^ level | Number of proteins |
| --- | --- |
| DNA helicase activity | 5 |
| Hydrogen ion transporting ATP synthase activity , rotational mechanism | 3 |
| ATP-dependant helicase activity | 2 |
| Sodium : amino acid symporter activity | 2 |
| Crossover junction endodeoxyribonuclease activity | 2 |
| ATP-dependent DNA helicase activity | 2 |
| DNA-dependent ATPase activity | 1 |
| DNA topoisomerase (ATP-hydrolyzing) activity | 1 |
| ATP-dependent peptidase activity | 1 |
| exodeoxyribonuclease I activity | 1 |
| hydrogen ion transmembrane transporter activity | 1 |
| ribonuclease III activity | 1 |
| bile acid:sodium symporter activity | 1 |
| UMP kinase activity | 1 |
| Type I site-specific deoxyribonuclease activity | 1 |

**Table S11** : Assignments of GO terms at the 9^th^ level
