# Supplementary material for: An automated approach for the identification of horizontal gene transfers from complete genomes reveals the rhizome of Rickettsiales
Source: BMC Evol Biol. 2012 Dec 12;12:243. doi: 10.1186/1471-2148-12-243 (PMC3575314; doi:10.1186/1471-2148-12-243)
Supplement: Additional file 1 — Table S1 shows the NCBI accession numbers for the studied species.Table S2 shows the Bayesian analysis for the horizontal gene transfer cases from other species to Rickettsiales excluding alphaproteobacteria. Violet rows represent transfer cases from at least 1 gain event, and the brown rows represent transfer cases from a non-orthologous group. PP indicates the posterior probability value from the Bayesian analysis, aa indicates amino acids, and BV indicates the bootstrap values obtained with the NJ, MP, and ML methods. Table S3 is a list of annotations for the conserved genes from 267 Ortholog clusters of Rickettsiales and Caulobacter cresentus CB15 from the COG database. Table S4 is a list of annotations for the conserved genes from 277 Ortholog clusters of Rickettsia, Orientia, Wolbachia, Anaplasma, Ehrlichia, Neorickettsia and Candidatus Pelagibacter ubique from the COG database. Table S5 is a list of annotation for the conserved genes from 328 Ortholog clusters of Rickettsia, Orientia, Wolbachia, Anaplasma, Ehrlichia and Neorickettsia from the COG database. Table S6 is a list of annotation sfor the 1,709 orthologous groups based on the COG database. Table S7 is a table of the horizontal gene transfer cases identified from at least 1 gain and 1 loss event (part 1). Table S8 is a table of the horizontal gene transfer cases identified from at least 1 gain and 1 loss event (part 2). Table S9 is a table of the horizontal gene transfer cases identified from non-orthologous groups. Table S10 is a table showing the distribution of proteins annotated from the Gene Ontology database. Table S11 is a table showing the assignment of proteins annotated by Gene Ontology at the 9th level. Table S12 is a table showing the assignment of proteins annotated by Gene Ontology at the 12th level. Table S13 is a table showing the non-synonymous/synonymous substitution analysis for de novo genes. [file 1471-2148-12-243-S1.ZIP › Additional data file 1 revised/Table S9.doc]

**TableS9. The cases of horizontal gene transfer identified from n**on-orthologous groups.

| **Sl.No** | **Gene identity number** | **Donor species** | **Recipient species** | **Protein annotation**  **from NCBI** | **Protein length**  **(aa)** | **BV**  **(NJ-MP-ML)** |
| --- | --- | --- | --- | --- | --- | --- |
| 1 | 238651169 | *Pseudomonas aeruginosa* PACS2  *Pseudomonas aeruginosa* PA7  (Gamma proteobacteria) | *Rickettsia peacockii* str. Rustic | hypothetical protein | 74 | 63-52-63 |
| 2 | 91205673 | *Anaerocellum thermophilum* DSM 6725  (Firmicutes) | Rickettsia bellii RML369-C | unnamed protein product | 540 | 100-100-99 |
| 3 | 71083820 | *Psychroflexus torquis* ATCC 700755 (Bacteroidetes) | *Candidatus Pelagibacter ubique* HTCC1062 | unnamed protein product | 133 | 100-100-74 |
| 4 | 71083857 | *Chlorobium ferrooxidans* DSM 13031 (Chlorobi)  NC10 bacterium 'Dutch sediment' (unclassified) | *Candidatus Pelagibacter ubique* HTCC1062 | unnamed protein product | 299 | 86-53-* |
| 5 | 67458540 & | *Yersinia frederiksenii* ATCC 33641  *Yersinia aldovae* ATCC 35236  *Yersinia ruckeri* ATCC 29473  *Yersinia kristensenii* ATCC 33638  *Yersinia rohdei* ATCC 43380  (Gamma proteobacteria) | *Rickettsia felis* URRWXCal2 | unnamed protein product | 257 | 98-99-* |
| 6 | 67458881 | *Cyanothece* sp. CCY0110  (Cyanobacteria) | *Rickettsia bellii* RML369-C  *Rickettsia conorii* str. Malish 7  *Rickettsia africae* ESF-5  *Rickettsia sibirica* 246  *Rickettsia rickettsii* str. Iowa  *Rickettsia rickettsii* str. 'Sheila Smith'  *Rickettsia massiliae* MTU5 | unnamed protein product | 79 | 51-99-* |
| 7 | 71083892 | *Shewanella violacea* DSS12  *Arcobacter butzleri* RM4018  *Grimontia hollisae* CIP101886  *Neptuniibacter caesariensis*  *Psychromonas ingrahamii* 37  *Moritella* sp. PE36  *Photobacterium profundum* 3TCK  *Vibrio* spp.  *Aeromonas salmonicida* subsp. salmonicida A449  *Aeromonas hydrophila* subsp. hydrophila ATCC 7966  *Shewanella* sp. MR-7  *Shewanella baltica OS185*  *Shewanella* sp. MR-4  (Gamma proteobacteria) | *Candidatus Pelagibacter* sp HTCC7211  *Candidatus Pelagibacter ubique* HTCC1062 | tcta gene product | 500 | 75-67-70 |
| 8 | 71083626 | *Arcobacter butzleri* RM4018  *Marinomonas* sp. MWYL1  *Psychromonas ingrahamii* 37  (Gamma proteobacteria) | *Candidatus Pelagibacter ubique* HTCC1062 | unnamed protein product | 632 | 96-66-* |
| 9 | 71083874 | *Bermanella marisrubri*  (Gamma proteobacteria) | *Alpha proteobacterium* HIMB114 *Candidatus pelagibacter ubique* HTCC1002 *Candidatus pelagibacter ubique* HTCC1062 *Candidatus pelagibacter* sp HTCC7211 | unnamed protein product | 124 | 90-73-* |
| 10 | 190571353 | *Bacillus* spp.  (Firmicutes) | *Wolbachia* endosymbiont of Culex quinquefasciatus Pel | glycosyl hydrolase family protein | 370 | 87-72-79 |
| 11 | 67459327 | *Kribbella flavida* DSM17836  (Actinobacteria) | *Rickettsia felis* URRWXCal2 | unnamed protein product | 97 | 99-91-87 |
| 12 | 71083893 | *Psychromonas ingrahamii* 37  *Moritella* sp. PE36  *Aliivibrio salmonicida* LFI1238  *Vibrio* spp.  *Photobacterium profundum* SS9  *Photobacterium profundum* 3TCK  (Gamma proteobacteria) | *Candidatus pelagibacter* sp. HTCC7211 *Candidatus pelagibacter ubique* HTCC1062 *Candidatus pelagibacter ubique* HTCC1002 | tctb gene product | 156 | 95-*-* |
| 13 | 71083901 | *Alkalilimnicola ehrlichii* MLHE-1  *Dictyoglomus turgidum* DSM 6724  *Dictyoglomus thermophilum* H-6-12  *Thermoanaerobacter tengcongensis* MB4  *Hyperthermus butylicus* DSM 5456  *Pyrobaculum calidifontis* JCM 11548  *Thermosphaera aggregans* DSM 11486  *Staphylothermus hellenicus* DSM 12710  *Staphylothermus marinus* F1  *Thermofilum pendens* HRK 5  *Caldivirga maquilingensis* JC-167  *Lyngbya* sp. PCC 8106  Malus sylvestrisarabidopsis halleri subsp. gemmifera  *Arabidopsis thaliana*  *Solanum lycopersicum*  *Cryptomeria japonica*  *Taxodium distichum*  *candidatus kuenenia stuttgartiensis*  *Sulfolobus tokodaii* str. 7  *Metallosphaera sedula* DSM 5348  *Sulfolobus solfataricus* P2  *Sulfolobus acidocaldarius* DSM 639  *Micromonas* sp. RCC299  *Ostreococcus lucimarinus* CCE9901  *Carboxydothermus hydrogenoformans* z-2901  *Prochlorococcus* spp.  *Candidatus korarchaeum cryptofilum* OPF8  *Francisella tularensis* subsp. holarctica lvs  *Clostridium perfringens* str.13  *Plasmodium yoelii yoelii* str. 17XNL  *Plasmodium berghei* str. Anka  *Plasmodium chabaudi* chabaudi  *Plasmodium falciparum* 3d7  *Oryza sativa japonica* group  *Anopheles gambiae* str. Pest  *Pediculus humanus* corporis  *Monosiga brevicollis MX1*  *Desulfitobacterium hafniense* DCB-2  *Desulfitobacterium hafniense* Y51  *Methanocaldococcus jannaschii* DSM 2661  *Symbiobacterium thermophilum* IAM14863  *Desulfotomaculum reducens* MI-1  *Picrophilus torridus* DSM 9790  *Thermoplasma volcanium* GSS1  *Thermoplasma acidophilum* DSM1728  *Ferroplasma acidarmanus* FER1 | *Candidatus pelagibacter* sp. HTCC7211 *Candidatus pelagibacter* *ubique* HTCC1002 *Candidatus pelagibacter ubique* HTCC1062 | spee gene product | 346 | 100-17-* |
| 14 | 225630935 | *Wolbachia* phage WO | *Wolbachia* sp. wRi  *Wolbachia* endosymbiont of Drosophila ananassae | Transposase | 444 | 100-*-* |
| 15 | 71083916 | *Hydra magnipapillata*  (Metazoa) | *Candidatus Pelagibacter* sp. HTCC7211  *Candidatus Pelagibacter ubique* HTCC1062 | unnamed protein product | 317 | 86-100-* |
| 16 | 42520763 | *Sulfolobus sp*. Y99.9.16  *Sulfolobus islandicus* Y.G.57.14  *Sulfolobus* sp. Y00.58.82  *Sulfolobus islandicus* Y.N.15.51  *Sulfolobus solfataricus* P2  *Sulfolobus solfataricus* 98.2  *Ferroplasma acidarmanus* FER1 (Archaea) | *Wolbachia* endosymbiont of Drosophila simulans  *Wolbachia* endosymbiont of Drosophila ananassae  *Wolbachia* endosymbiont of Drosophila melanogaster  *Wolbachia* endosymbiont of Drosophila willistoni tsc#14030-0811 24  *Wolbachia* endosymbiont of Culex quinquefasciatus jhb | is5 family transposase | 135 | 92-*-* |
| 17 | 71084007 & | *Synechococcus* sp. WH7803  *Prochlorococcus marinus* str. MIT 9515  *Prochlorococcus marinus* subsp. Pastoris str. CCMP1986  *Prochlorococcus marinus* str. MIT 9312  *Prochlorococcus marinus* str. MIT 9301  *Prochlorococcus* marinus str. AS9601  *Prochlorococcus marinus* str. MIT 9202  *Prochlorococcus marinus* str. MIT 9215  *Prochlorococcus marinus* str. MIT 9211  (Cyanobacteria) | *Candidatus Pelagibacter ubique* HTCC1062  *Candidatus Pelagibacter* sp. HTCC7211 | unnamed protein product | 139 | 87-45-55 |
| 18 | 71083252 | *Dickeya zeae* ECH1591  *Photobacterium* sp. SKA34  *Psychromonas* sp. CNPT3  *Helicobacter cinaedi* CCUG18818  *Helicobacter canadensis* MIT98-5491  *Campylobacter gracilis* RM3268  *Desulfobulbus propionicus* DSM 2032  *Vibrio* sp. RC341  *Burkholderia* sp. CCGE1001  *Azoarcus s*p. BH72  *Brachyspira murdochii* DSM 12563  *Flavobacteria bacterium* BAL38  *Oribacterium sinus* F0268  *Butyrivibrio proteoclasticus* B316  *Anaerotruncus colihominis* DSM 17241  *Clostridium hathewayi* DSM13479  *Leptotrichia goodfellowii* F0264  *Bacteroides xylanisolvens* XB1A  *Bacteroides* sp. 1_1_30 (Gammaproteobacteria, Betaproteobacteria, Bacteroidetes) | *Candidatus pelagibacter ubique* HTCC1062 | unnamed protein product | 329 | 97-*-* |

(&) indicates the HGT cases reported from previous studies
