# Supplementary material for: An automated approach for the identification of horizontal gene transfers from complete genomes reveals the rhizome of Rickettsiales
Source: BMC Evol Biol. 2012 Dec 12;12:243. doi: 10.1186/1471-2148-12-243 (PMC3575314; doi:10.1186/1471-2148-12-243)
Supplement: Additional file 1 — Table S1 shows the NCBI accession numbers for the studied species.Table S2 shows the Bayesian analysis for the horizontal gene transfer cases from other species to Rickettsiales excluding alphaproteobacteria. Violet rows represent transfer cases from at least 1 gain event, and the brown rows represent transfer cases from a non-orthologous group. PP indicates the posterior probability value from the Bayesian analysis, aa indicates amino acids, and BV indicates the bootstrap values obtained with the NJ, MP, and ML methods. Table S3 is a list of annotations for the conserved genes from 267 Ortholog clusters of Rickettsiales and Caulobacter cresentus CB15 from the COG database. Table S4 is a list of annotations for the conserved genes from 277 Ortholog clusters of Rickettsia, Orientia, Wolbachia, Anaplasma, Ehrlichia, Neorickettsia and Candidatus Pelagibacter ubique from the COG database. Table S5 is a list of annotation for the conserved genes from 328 Ortholog clusters of Rickettsia, Orientia, Wolbachia, Anaplasma, Ehrlichia and Neorickettsia from the COG database. Table S6 is a list of annotation sfor the 1,709 orthologous groups based on the COG database. Table S7 is a table of the horizontal gene transfer cases identified from at least 1 gain and 1 loss event (part 1). Table S8 is a table of the horizontal gene transfer cases identified from at least 1 gain and 1 loss event (part 2). Table S9 is a table of the horizontal gene transfer cases identified from non-orthologous groups. Table S10 is a table showing the distribution of proteins annotated from the Gene Ontology database. Table S11 is a table showing the assignment of proteins annotated by Gene Ontology at the 9th level. Table S12 is a table showing the assignment of proteins annotated by Gene Ontology at the 12th level. Table S13 is a table showing the non-synonymous/synonymous substitution analysis for de novo genes. [file 1471-2148-12-243-S1.ZIP › Additional data file 1 revised/Table S8.doc]

**Table S8. The cases of horizontal gene transfer identified from at least 1 gain and 1 loss event (part2).**

| **Sl.No** | **Gene identity number** | **Donor species** | **Recipient species** | **Protein annotation**  **from NCBI** | **Protein length**  **(aa)** | **BV**  **(NJ_MP_ML)** |
| --- | --- | --- | --- | --- | --- | --- |
| 1 | 42520378 & | *Aedes aegypti*  (Arthropoda) | *Wolbachia* endosymbiont of Drosophila melanogaster  *Wolbachia* endosymbiont of Culex quinquefasciatus Pel | Hypothetical protein | 2843 | 100-100-100 |
| 2 | 190570783 & | *Wolbachia* phage WO | *Wolbachia* endosymbiont of Culex quinquefasciatus pel  *Wolbachia* endosymbiont of Drosophila melanogaster  *Wolbachia* endosymbiont of Drosophila willistoni tsc#14030-0811 24  *Wolbachia* endosymbiont of Drosophila ananassae | Putative phage related protein | 143 | 100-100-95 |
| 3 | 91205671 | *Vibrio* spp.  *Shewanella* spp.  *Providencia* spp.(Gamma proteobacteria)  *Lactobacillus* spp.  *Bacillus* spp.  *Listeria* spp.  *Enterococcus* spp. (Firmicutes) | *Rickettsia bellii* OSU 85-389  *Rickettsia bellii* RML 369-C  *Rickettsia canadensis* str. McKiel  *Rickettsia akari* str. Hartford  *Rickettsia felis* URRWXCal2  *Rickettsia typhi* str. Wilmington  *Rickettsia prowazenkii* RP22  *Rickettsia prowazenkii* str. Madrid E | MFS-type bicyclomycin resistance protein | 404 | 87-61-* |
| 4 | 238651161 | *Actinobacillus minor* NM305  (Gamma proteobacteria) | *Rickettsia felis* URRWXCal2  *Rickettsia peacockii* str. Rustic | Hypothetical protein | 82 | 48-96-* |
| 5 | 189182975 | *Francisella novicida* GA99-3548  *Francisella tularensis* subsp. holarctica  *Francisella tularensis* subsp. holarctica OSU18  *Francisella tularensis* subsp. tularensis SCHU S4  *Francisella tularensis* subsp. mediasiatica FSC147  *Francisella novicida* GA99-3549  *Francisella novicida* U112  *Francisella novicida* FTG  (Gamma proteobacteria) | *Orientia tsutsugamushi* str. Boryong  *Orientia tsutsugamushi* str. Ikeda  *Wolbachia* endosymbiont of Culex quinquefasciatus Pel | Hypothetical protein | 632 | 100-95-* |
| 6 | 157826324 | *Candidatus Nitrospira* defluvii  (Nitrospirales) | *Rickettsia akari* str. Hartford  *Rickettsia bellii* OSU 85-389  *Rickettsia bellii* RML 369-C | Growth inhibitor | 88 | 99-*-100 |
| 7 | 157964120 | *Bacterium* ellin514  *Bacteroide*s sp. 1_1_30  *Bacteroides eggerthii* 1_2_48faa  *Bacteroides eggerthii* DSM 20697  *Bacteroides stercoris* ATCC 43183  *Alistipes indistinctus* YIT 12060 (Firmicutes) | *Rickettsia bellii* RML 369-C  *Rickettsia canadensis* str Mckiel  *Rickettsia* endosymbiont of Ixodes scapularis  *Rickettsia felis* URRWXCal2  *Rickettsia japonica* YH  *Rickettsia sibirica* 246  *Rickettsia africae* ESF-5  *Rickettsia peacockii* str. Rustic  *Rickettsia conorii* str. Malish 7  *Rickettsia rickettsii* str. 'Sheila Smith'  *Rickettsia massiliae* Mtu5  *Rickettsia akari str*. Hartford  *Rickettsia prowazekii* str. Madrid E  *Rickettsia typhi* str. Wilmington | Sugar phosphate permease | 439 | 92-100-* |
| 8 | 157827290 | *Candidatus Nitrospira defluvii*  (Nitrospirales) | *Rickettsia felis* URRWXCal2  *Rickettsia bellii* OSU 85-389  *Rickettsia bellii* RML369-C | Hypothetical protein | 74 | 100-*-* |
| 9 | 157964562 | *Bacillus tusciae* DSM 2912 | *Rickettsia massiliae* MTU5  *Rickettsia conorii* str. Malish 7  *Rickettsia africae* ESF-5  *Rickettsia felis* URRWXCal2 | Antitoxin of toxin-antitoxin | 50 | 100-*-* |
| 10 | 157827155 | *Treponema denticola* F0402  (Spirochatetes) | *Rickettsia felis* URRWXCal2  *Rickettsia belli* *i* OSU 85-389  *Rickettsia belli i* RML369-C | Hypothetical protein | 94 | 83-55-80 |

(&) indicates HGT cases reported in previous studies.
