# Supplementary material for: An automated approach for the identification of horizontal gene transfers from complete genomes reveals the rhizome of Rickettsiales
Source: BMC Evol Biol. 2012 Dec 12;12:243. doi: 10.1186/1471-2148-12-243 (PMC3575314; doi:10.1186/1471-2148-12-243)
Supplement: Additional file 1 — Table S1 shows the NCBI accession numbers for the studied species.Table S2 shows the Bayesian analysis for the horizontal gene transfer cases from other species to Rickettsiales excluding alphaproteobacteria. Violet rows represent transfer cases from at least 1 gain event, and the brown rows represent transfer cases from a non-orthologous group. PP indicates the posterior probability value from the Bayesian analysis, aa indicates amino acids, and BV indicates the bootstrap values obtained with the NJ, MP, and ML methods. Table S3 is a list of annotations for the conserved genes from 267 Ortholog clusters of Rickettsiales and Caulobacter cresentus CB15 from the COG database. Table S4 is a list of annotations for the conserved genes from 277 Ortholog clusters of Rickettsia, Orientia, Wolbachia, Anaplasma, Ehrlichia, Neorickettsia and Candidatus Pelagibacter ubique from the COG database. Table S5 is a list of annotation for the conserved genes from 328 Ortholog clusters of Rickettsia, Orientia, Wolbachia, Anaplasma, Ehrlichia and Neorickettsia from the COG database. Table S6 is a list of annotation sfor the 1,709 orthologous groups based on the COG database. Table S7 is a table of the horizontal gene transfer cases identified from at least 1 gain and 1 loss event (part 1). Table S8 is a table of the horizontal gene transfer cases identified from at least 1 gain and 1 loss event (part 2). Table S9 is a table of the horizontal gene transfer cases identified from non-orthologous groups. Table S10 is a table showing the distribution of proteins annotated from the Gene Ontology database. Table S11 is a table showing the assignment of proteins annotated by Gene Ontology at the 9th level. Table S12 is a table showing the assignment of proteins annotated by Gene Ontology at the 12th level. Table S13 is a table showing the non-synonymous/synonymous substitution analysis for de novo genes. [file 1471-2148-12-243-S1.ZIP › Additional data file 1 revised/Table S7.doc]

**Table S7. The** cases of horizontal gene transfer identified from at least 1 gain and 1 loss event (part1).

| **Sl.No** | **Gene identity number** | **Donor species** | **Recipient species** | **Protein annotation**  **from NCBI** | **Protein length**  **(aa)** | **BV**  **(NJ_MP_ML)** |
| --- | --- | --- | --- | --- | --- | --- |
| 1 | 157964915 | *Candidatus Amoebophilus asiaticus* 5a2  (Bacteroidetes) | *Rickettsia bellii* RML369-C  *Rickettsia peacockii* str. Rustic  *Rickettsia rickettsii* str. Sheila Smith  *Rickettsia massiliae* Mtu5 | ATPase | 384 | 75-*-63 |
| 2 | 157825906 | *Legionella drancourtii* LLAP12  (Gamma proteobacteria) | *Rickettsia felis* URRWXCal2  *Rickettsia akari* str. Hartford | Unnamed product protein | 104 | 90-*-* |
| 3 | 157827021 | *Candidatus Amoebophilus asiaticus* 5a2  (Bacteroidetes) | *Rickettsia akari* str. Hartford  *Rickettsia bellii* OSU 85-389  *Rickettsia bellii* RML 369-C | Unnamed product protein | 130 | 99-90-50 |
| 4 | 157827012 | *Frankia* sp. CCI3  *Stigmatella aurantiaca* DW4 3-1  (Delta proteobacteria) | *Rickettsia bellii* OSU 85-389  *Rickettsia bellii* RML 369-C | Unnamed product protein | 278 | 98-84-82 |
| 5 | 157826843 | *Haemophilus parasuis* 29755  *Leeuwenhoekiella blandensis* MED21  *Haemophilus influenzae* pittee  *Haemophilus influenzae* R2846  *Haemophilus influenzae* 7p49h1  *Parabacteroides distasonis* ATCC 8503  (Gamma proteobacteria) | *Rickettsia bellii* OSU 85-389  *Rickettsia bellii* RML 369-C | Unnamed product protein | 202 | 99-*-* |
| 6 | 225630595 | *Francisella tularensis*  (Gamma-proteobacteria) | *Wolbachia* endosymbiont of Culex | Dihydroneopterin aldolase | 128 | 100-73-98 |
| 7 | 254797178 | *Candidatus protochlamydia amoebophila*  *Parachlamydia acanthamoebae* str. Hall ‘s coccus  (Chlamydia) | *Neorickettsia sennetsu* str. Miyayama  *Neorickettsia sennetsu* str. Illinois | Branched-chain amino acid  transporter | 380 | 98-100-* |
| 8 | 56416493 | *Lawsonia intracellularis* Phe/MN1-00  (Deltaproteobacteria) | *Anaplasma marginale* str. St. Maries | Hypothetical protein | 370 | 98-67-75 |
| 9 | 157827797 | *Aminomonas paucivorans* DSM 12260  (Synergistetes)  *Leptospirillum ferrodiazotrophum* (Nitrospirales)  *Verminephrobacter* sp. AT4  (Beta proteobacteria)  *Xylella fastidiosa* 9A5C  (Gamma proteobacteria) | *Rickettsia akari* str. Hartford  *Rickettsia bellii* OSU 85-389  *Rickettsia bellii* RML 369-C | PemK-like growth inhibitor | 108 | 93-60-64 |
| 10 | 225630592 | *Chlamydophila pneumoniae* CWL029  *Chlamydophila pneumoniae* AR39  *Chlamydophila pneumoniae* J138  *Chlamydia trachomatis* 70  *Chlamydia trachomatis DS* 2923  *Chlamydia trachomatis* 434 bu  *Chlamydia trachomatis* d uw-3 cx  *Chlamydia muridarum* Weiss  *Chlamydia muridarum* Nigg  *Chlamydophila abortus* s26-3  *Chlamydophila caviae* gpic  *Chlamydophila felis* fe c-56 | *Wolbachia* endosymbiont of Drosophila melanogaster  Wolbachia endosymbiont of Drosophila ananassae  Wolbachia sp. wRi | Hypothetical protein | 251 | 91-53-72 |
| 11 | 225630748 | *Pseudomonas fluorescence* SB W25  *Plesiocystis pacifica* SI R1  *Haliangium ochraceum DSM* 143-65  (Gamma proteobacteria) | *Rickettsia* endosymbiont of Ixodes scapularis  *Wolbachia* sp. wRi  *Wolbachia* endosymbiont of Drosophila simulans | Hypothetical protein | 329 | 100-100-98 |
| 12 | 225630750 | *Geobacillus kaustophilus* HTA426  *Geobacillu*s sp. C56-T3  *Anoxybacillus flavithermus* WK1  *Bacillus thuringiensis serovar andalousiensis* bgsc 4AW1  *Bacillus cereus* rock1-3  (Firmicutes) | *Rickettsia* endosymbiont of Ixodes scapularis  *Wolbachia* endosymbiont of Drosophila melanogaster  Wolbachia sp. wRi  Wolbachia endosymbiont of Drosophila willistoni TSC#14030-0811 24 | L-allo-threonine aldolase putative | 349 | 97-74-85 |
| 13 | 67459358 | *Burkholderia xenovorans* LB400  (Beta proteobacteria) | *Rickettsia felis* URRWXCal2  *Rickettsia* endosymbiont of Ixodes scapularis | Transposase | 281 | 95-99-* |
| 14 | 229586611 | *Candidatus Amoebophilus asiaticus* 5a2  (Bacteroidetes) | *Rickettsia africae* ESF-5  *Rickettsia japonica* YH  *Rickettsia peacockii* str. Rustic  *Rickettsia rickettsii* str. Iowa  *Rickettsia rickettsii* str. Sheila Smith  *Rickettsia sibirica* 246  *Rickettsia felis* URRWXCal2 | Unnamed protein | 209 | 100-98-* |
| 15 | 157826681 | *Dictyostelium discoideum AX4*  *Polysphondylium pallidum* PN500  (Protozoa) | *Rickettsia felis* URRWXCal2  *Rickettsia bellii* OSU 85-389  *Rickettsia bellii* RML 369-C | Unnamed protein product | 113 | 100-*-100 |
